# Structure and multipartite genome architecture of the mitochondrial genome in the endangered medicinal plant *Fritillaria taipaiensis* P. Y. Li

**DOI:** 10.3389/fsysb.2026.1708877

**Published:** 2026-04-22

**Authors:** De Xu, Jingtao Liu, Juan Huang, Qianqian Ma, Tao Wang, Zhou Xie, Xue Liu, Liang Fu

**Affiliations:** 1 Institute of Chinese Materia Medica, Dazhou Academy of Agricultural Sciences, Dazhou, China; 2 Dazhou Key Laboratory of Agricultural Resources Development and Ecological Conservation in Daba Mountain, Sichuan University of Arts and Science, Dazhou, China; 3 Chongqing Key Laboratory of Traditional Chinese Medicine Resource, Endangered Medicinal Breeding National Engineering Laboratory, Chongqing Academy of Chinese Materia Medica, Chongqing, China

**Keywords:** endangered medicinal plant, Fritillaria taipaiensis, genetransfer, multiple circular mitochondrial genome, RNA editing events

## Abstract

Fritillaria taipaiensis: a medicinal plant of the Liliaceae family endemic to the Qinling Mountains and southern regions of China, is renowned for its therapeutic properties in relieving cough and moistening the lungs. Despite growing interest in the organellar genomes of related species, the mitochondrial genome (mitogenome) of *F. taipaiensis* has remained uncharacterized. In this study, we successfully assembled and annotated the complete mitogenome of *F. taipaiensis* using a combination of Illumina and Nanopore technologies. The mitogenome consisted of 23 circular chromosomes with a total length of 847,160 bp and contained 49 annotated genes, including 34 protein-coding genes (PCGs), 12 transfer RNA (tRNA) genes, and 3 ribosomal RNA (rRNA) genes. Codon usage analysis revealed a strong bias toward A- and U-ending codons. Additionally, we identified 178 simple sequence repeats (SSRs) and 127 dispersed repeats. We also detected ten chloroplast-derived DNA fragments transferred to the mitogenome, most of which involved tRNA genes. A total of 488 RNA editing events were predicted in PCGs, all involving C-to-U conversions. Phylogenetic analysis based on 21 representative plant mitogenomes placed *F. taipaiensis* in a position congruent with its traditional taxonomic classification. These findings enhance our understanding of the mitogenome architecture and evolutionary dynamics of *F. taipaiensis* and provide valuable genomic resources for future studies in conservation biology, population genetics, and comparative genomics within the *Fritillaria* genus.

## 1 Introduction

The genus *Fritillaria* (Liliaceae) comprises approximately about 168 perennial herbaceous species distributed across temperate regions of the Northern hemisphere, ranging from Eurasia to Northern Myanmar, Alaska to Northwest Mexico, and Central Asia to China and Japan (https://powo.science.kew.org/, accessed on 9 July 2025). Among the 24 species recorded in China, 15 are endemic, and many most possessing significant medicinal and economic value (Wu et al., 2020). *Fritillaria taipaiensis* P. Y. Li, commonly known as “taibei” or “jianbei”, is mainly distributed in Sichuan, Chongqing, Shanxi, and Hubei provinces of China, where it grows on grassy hillsides or near water bodies at altitudes of 1800–3,150 m (Hu et al., 1993). Compared with other medicinal *Fritillaria* species, such as *F. cirrhosa*, which typically occurs above 3,000 m, *F. taipaiensis* is more suitable for cultivation at relatively lower altitudes. However, due to long-term overexploitation and environmental changes, natural populations of *F. taipaiensis* have declined dramatically, and the species has been listed as a national second-grade endangered medicinal plant in China, highlighting the need for comprehensive genetic studies to support its conservation and utilization.

In recent years, genomic research on *F. taipaiensis* has primarily focused on chloroplast genome characterization, molecular marker development, and genetic diversity analysis (Xu et al., 2024; Xie et al., 2024). The complete chloroplast genome of *F. taipaiensis* is 151,693 bp in length and exhibits the typical quadripartite structure, consisting of a large single-copy region (LSC), a small single-copy region (SSC), and a pair of inverted repeat regions (IRs). Several mutation hotspots have also been identified, providing potential targets for molecular marker development and insights into long-term adaptation to alpine environments (Xu et al., 2024). Besides, among the *Fritillaria* genus, the mitogenome of *F. ussuriensis* was sequenced and assembled only, and revealing 13 circular chromosomes (Xie et al., 2024). The limited availability of mitochondrial genomic data hinders a comprehensive understanding of phylogenetic relationships, evolutionary patterns, and conservation genetics within the genus.

Mitochondria are essential eukaryotic organelles containing their own genetic systems and typically exhibit maternal inheritance (Birky, 2001). Plant mitochondrial genomes are structurally complex and highly variable in size, ranging from 66 Kb to 11.3 Mb across species (Liu et al., 2024). This complexity is largely attributed to frequent recombination events, intracellular gene transfer (IGT), and horizontal gene transfer (HGT), which contribute to mitochondrial genome plasticity (Gandini and Sanchez-Puerta, 2017; Zhao et al., 2018; Kan et al., 2020). Advances in long-read sequencing technologies have greatly facilitated the assembly of complex organelle genomes, enabling more accurate characterization of mitochondrial genome (mitogenome) structures (Milne et al., 2013).

Mitogenome analysis is essential for understanding plant evolution and genomic structure. Up to now, the mitogenome of *F. taipaiensis* has not yet been sequenced and assembled. Thus, characterizing its mitogenome and conducting comparative and evolutionary analysis is crucial for enhancing our understanding of its taxonomic and evolutionary context within the genus *Fritillaria.* In this study, we aimed to elucidate the structural features and genomic architecture of the mitogenome of *F. taipaiensis*, an endangered medicinal plant with limited genomic resources. Specifically, we sought to clarify the multipartite organization, structural characteristics, and evolutionary position of its mitogenome within angiosperms. By generating and analyzing the complete mitogenome sequence, this study provides a genomic foundation for understanding mitogenome evolution in *Fritillaria* and supports future conservation and genetic improvement efforts for this endangered species.

## 2 Materials and methods

### 2.1 Plant materials, sequencing and quality control

Freshly collected cultivated specimens of *F. taipaiensis* were collected from the Hua’e Mountain National Nature Reserve in Dazhou City, Sichuan Province, China (E 108°00′–108°27′, N 31°55′–31°12′). All samples were taxonomically identified by Prof. Hao Zhang (West China School of Pharmacy, Sichuan University), and voucher specimens have been deposited at the Dazhou Academy of Agricultural Sciences. Total genomic DNA was extracted using the cetyltrimethylammonium bromide (CTAB) protocol (Doyle and Doyle, 1987). The mitogenome of *F. taipaiensis* was sequenced using Illumina and Nanopore technologies, respectively. For Illumina sequencing, 1 ng of DNA was used to construct a short-read library (average insert size of 350 bp), which was sequenced on the DNBseq platform (California, United States of America). For Oxford Nanopore sequencing, sequence libraries were generated using the SQK-LSK109 ligation kit following the manufacturer’s protocols. The prepared library was then loaded onto primed R9.4 Spot-on Flow Cells and sequenced using a PromethION sequencer (Oxford Nanopore Technologies, Oxford, UK) over 48-h runs. Base calling was performed using the standard ONT base-calling pipeline provided by the sequencing platform, and the resulting base-called reads were used for all subsequent analyses. Sequencing yielded 2.9 M ONT reads (34.2 Gb; mean length ∼11.8 Kb) and 115.4 M Illumina reads (34.6 Gb), ensuring adequate long- and short-read coverage for high-quality assembly of the mitogenome. Illumina reads were trimmed using fastp v 0.23.4 (Chen et al., 2018) (default settings) to remove adapters and low-quality bases.

### 2.2 Assembly and annotation of organelle genomes

To ensure accurate assembly of the *F. taipaiensis* mitogenome, a hybrid sequencing strategy combining Illumina short reads and Nanopore long reads was used. The following files have been checked extensively for errors.

The mitogenome of *F. taipaiensis* was initially assembled from long-read sequencing data using Flye (2.9.1-b1780) with default parameters, generating graphical fragment assembly (GFA) files. All assembled contigs in FASTA format were compiled into a local BLAST database, and mitochondrial genes from *F. ussuriensis* (OR783162.1- OR783174.1) and plant conserved mitochondrial genes dataset were used as query sequences in BLASTn to identify contigs containing mitochondrial genes with the parameters: -evalue 1e-5 -outfmt 6 -max_hsps 10 -word_size 7 -task blastn-short. Subsequently, quality-filtered Illumina short reads were aligned to the putative mitochondrial contigs using BWA-MEM v0.7.17 with default parameters, (Li and Durbin, 2009), and Pilon v1.24 was run for three rounds to correct residual single-base errors and small indels using only Illumina reads. The annotation of protein-coding genes (PCGs) was conducted with Geseq website (https://chlorobox.mpimp-golm.mpg.de/geseq.html) (Tillich et al., 2017) (version 2.03) and PMGA tool (http://www.1kmpg.cn/pmga/) (Li J. et al., 2024) using four mitogenomes as reference, including *F. ussuriensis* (OR783162- OR783174), *Lilium davidii* (MT985331-MT985346), *L*. *tsingtauense* (OP973783-OP973810), *L*. *pumilum* (MT985347-MT985363). In addition, the PMGA dataset contains a curated set of core mitochondrial genes conserved across angiosperms, which was also used as a reference for annotation. Transfer RNA (tRNA) and ribosomal RNA (rRNA) within the mitogenome were annotated using the tRNAscan-SE (version 2.0.11) (Lowe and Eddy, 1997) and BLASTN software (version 2.13.0) (Chen et al., 2015), respectively. All annotation results were manually curated using Apollo software (version 1.11.8) (Lewis et al., 2002). The Bandage software (version 0.8.1) (Wick et al., 2015) was used to visualize the graphical mitogenome results. The final assembly and annotation results were deposited in the National Center for Biotechnology Information (NCBI) database (https://www.ncbi.nlm.nih.gov/) under accession numbers: *F. taipaiensis* (PV819105–PV819127).

### 2.3 Repeat sequence analysis

For simple sequence repeat (SSR), tandem repeat, and dispersed repeat analyses, multiple tools were employed: MISA (version 2.1) (https://webblast.ipk-gatersleben.de/misa/) (parameters: 1–10, 2–5,3–4, 4–3, five to three, and 6–3) (Beier et al., 2017), the Tandem Repeats Finder (TRF, version 4.09) (https://tandem.bu.edu/trf/trf.unix.help.html) (parameters: default) (Benson, 1999), and the REPuter server (parameters: default) (https://bibiserv.cebitec.unibielefeld.de/reputer/) (Kurtz et al., 2001), respectively. Visualization of repeat elements was accomplished using the Circos package (version 0.69.9) (Zhang et al., 2013) and Excel 2021.

### 2.4 Codon usage bias analysis and RNA editing site analysis

PCGs were systematically extracted from the mitogenome assemblies using the PhyloSuite software (version1.1.16) (Zhang et al., 2020), which facilitates automated retrieval and management of gene datasets for phylogenomic analysis. To assess codon usage bias, relative synonymous codon usage (RSCU) values were calculated using the MEGA software (version 7.0) (Kumar et al., 2016). The effective number of codons (ENC) and the codon adaptation index (CAI) were calculated using DAMBE, ENC-GC3s plot were generated by R.

RNA editing site prediction for C-to-U conversions within the PCGs of the *F. taipaiensis* mitogenome was conducted using the Deepred-mt (Edera et al., 2021), a deep-learning-based prediction tool, with default parameters.

### 2.5 Phylogenetic analysis

To reconstruct the phylogenetic relationships within Liliaceae (Liliales), mitogenome sequences from 21 representative species (Table 1) were retrieved from the NCBI Organelle Genome Resources database (https://www.ncbi.nlm.nih.gov/genome/organelle/). Two species from the order Ranunculales, *Anemone maxima* (NC_053368.1) and *Aconitum kusnezoffii* (NC_053920.1), were used as outgroup. A total of 24 shared PCGs were identified through reciprocal BLASTp analysis across the retrieved mitogenomes. Multiple sequence alignments of the concatenated PCGs were performed using the MAFFT software (version 7.505) (Katoh et al., 2002) with the parameters: -auto. The multiple sequence alignment (MSA) generated in this study has been deposited in Figshare (http://doi.org/10.6084/m9.figshare.30939188). Phylogenetic inference was carried out using the IQ-TREE software (version 1.6.12) with the following parameters: -alrt 1000 -B 1000 (Nguyen et al., 2015). The resulting maximum likelihood tree was visualized using the ITOL software (version 4.0) (Letunic and Bork, 2019).

**TABLE 1:** The information of mitogenome in the phylogenetics analysis.

Order	Species	NCBI accession
Liliales	Fritillaria taipaiensis	PV819105-PV819127
​	Fritillaria usuriensis	OR783162_74.1
​	Lilium davidii	MT985331_46.1
​	Lilium pumilum	MT985347_63.1
​	Lilium tsingtauense chromosome 1–28 mitochondrion, complete sequence	OP973783_810.1
Arecales	Cocos nucifera mitochondrion, complete genome	NC_031696.1
​	Phoenix dactylifera mitochondrion, complete genome	NC_016740.1
Alismatales	Pinellia ternata mitochondrion, complete genome	OQ948332.1
​	Zantedeschia aethiopica mitochondrion, complete sequence	NC_073008.1
​	Zostera japonica mitochondrion, complete genome	NC_068803.1
​	Butomus umbellatus mitochondrion, complete genome	NC_021399.1
​	Stratiotes aloides voucher Seberg et al., C2459 mitochondrion, complete genome	NC_035317.1
​	Spirodela polyrhiza mitochondrion, complete genome	NC_017840.1
Asparagales	Crocus sativus mitochondrion, complete genome	OL804177.1
​	Chlorophytum comosum mitochondrion, complete genome	MW411187.1
​	Asparagus officinalis cultivar Atlas mitochondrion, complete genome	NC_053642.1
​	Allium cepa mitochondrion, complete genome	NC_030100.1
​	Hemerocallis citrina mitochondrion, complete sequence	MZ726801_3.1
​	Apostasia shenzhenica mitochondrion, complete genome	OQ645347.1
Ranunculales	Aconitum kusnezoffii mitochondrion, complete genome	NC_053920.1
​	Anemone maxima mitochondrion, complete genome	NC_053368.1

### 2.6 Identification of homologous fragment and collinear analysis

The chloroplast genome of *F. taipaiensis* was assembled using GetOrganelle (version 1.7.5) with the following parameters: -R 10; -F embplant_pt. Following assembly, annotation of the chloroplast genome was performed using the CPGAVAS2 software (version 2.0) (Shi et al., 2019). To investigate homologous sequences between the chloroplast and mitochondrial genomes were identified using the BLASTn software (version 2.13.0) with parameter: e-value 1e−6 word size 7, and the results were visualized using the Circos package (version 0.69.9). Additionally, the mitogenome pairwise comparison was conducted using BLASTN (parameters: -evalue 1e-5, -word_size 9, -gapopen 5, -gapextend 2, -reward 2, -penalty −3). Homologous sequences longer than 0.5 Kb were used to construct conserved collinearity blocks in the multiple synteny plot, which was visualized using MCScanX (Wang et al., 2012). Comparative synteny analysis was conducted between *F. taipaiensis* and four related species: *Fritillaria usuriensis* (OR783162-OR783174), *L. davidii* (MT985331- MT985346), *L. tsingtauense* (OP973783-OP973810), and *L. pumilum* (MT985347-MT985363).

## 3 Results

### 3.1 General features of the *F. taipaiensis* mitogenome

**FIGURE 1:**
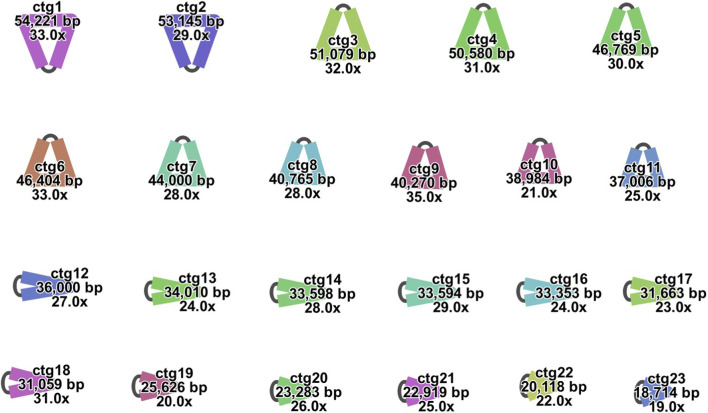
Circular representation of the mitochondrial genome assembly of F. taipaiensis. The figure shows the assembly result visualized in Bandage, display-ing 23 circular contigs.

The mitogenome of *F. taipaiensis* consists of 23 circular contigs, exhibiting a multi-circular structure, collectively spanning 847,160 bp with a GC content of 44.98% (Figure 1A). Based on the *de novo* assembly results, a total of 2,911,201 quality-filtered long reads were obtained, of which 8,291 supported the mitogenome assembly, 35,954 supported the chloroplast genome assembly, and the remaining 2,866,956 reads were most likely derived from the nuclear genome. The complete mitogenome was available in GenBank under accession numbers PV819105–PV819127, and the length, GC content, accession information, and mapping results for each chromosome are summarized in Table 2 and Table 3. The sequencing depth and coverage profiles for the mitogenome are provided in Supplementary Figures S1–S3. Although the average ONT read length is approximately 12 Kb (Supplementary Tables S1,S2), individual long reads frequently align only partially to the mitogenome, resulting in coverage peaks that are shorter than the full read length. These peaks are generated by the accumulation of overlapping partial alignments from multiple long reads. Notably, many of these coverage peaks coincide with regions identified as MTPTs. In addition, read coverage patterns at contig termini provide supporting evidence for chromosome circularization (Supplementary Figures S4–S6). Distinct and continuous coverage signals were observed across the terminal regions of several contigs, consistent with circular mitochondrial chromosomes rather than linear structures.

**TABLE 2:** The information of the *F. taipaiensis* mitogenome.

Type	Structure	NCBI accession number	Total length (bp)	GC content (%)	Depth (×)
Mitochondrial genome	Multiple branched	PV819105-PV819127	847,160	44.98	27
Chromosome l	Circular	PV819105	54,221	45.44	33
Chromosome 2	Circular	PV819106	53,145	45.5	29
Chromosome 3	Circular	PV819107	51,079	44.57	32
Chromosome 4	Circular	PV819108	50,580	44.91	31
Chromosome 5	Circular	PV819109	46,769	44.81	30
Chromosome 6	Circular	PV819110	46,404	45.1	33
Chromosome 7	Circular	PV819111	44,000	45.24	28
Chromosome 8	Circular	PV819112	40,765	45.1	28
Chromosome 9	Circular	PV819113	40,270	44.61	35
Chromosome 10	Circular	PV819114	38,984	44.33	21
Chromosome 11	Circular	PV819115	37,006	44.58	25
Chromosome 12	Circular	PV819116	36,000	44.66	27
Chromosome 13	Circular	PV819117	34,010	44.93	24
Chromosome 14	Circular	PV819118	33,598	44.69	28
Chromosome 15	Circular	PV819119	33,594	44.55	29
Chromosome 16	Circular	PV819120	33,353	44.71	24
Chromosome 17	Circular	PV819121	31,663	45.75	23
Chromosome 18	Circular	PV819122	31,059	44.52	31
Chromosome 19	Circular	PV819123	25,626	46.02	20
Chromosome 20	Circular	PV819124	23,283	45.34	26
Chromosome 21	Circular	PV819125	22,919	44.02	25
Chromosome 22	Circular	PV819126	20,118	46.28	22
Chromosome 23	Circular	PV819127	18,714	45.55	19

**TABLE 3:** Nanopore and Illunina mapping results in the mitogenome of *F. taipaiensis.*

Chromosome	Illumina mapping mean depth (×)	ONT mapping mean depth (×)
Chromosome 1	277.05	275.916
Chromosome 2	158.656	166.061
Chromosome 3	192.883	224.142
Chromosome 4	182.593	193.435
Chromosome 5	130.077	136.428
Chromosome 6	127.55	120.107
Chromosome 7	112.04	104.305
Chromosome 8	146.998	180.768
Chromosome 9	141.798	125.653
Chromosome 10	100.496	97.3028
Chromosome 11	120.602	108.663
Chromosome 12	104.06	97.5196
Chromosome 13	112.715	133.273
Chromosome 14	96.5613	101.997
Chromosome 15	113.121	109.082
Chromosome 16	119.221	117.981
Chromosome 17	104.114	145.576
Chromosome 18	129.925	122.197
Chromosome 19	107.559	125.064
Chromosome 20	125.074	111.016
Chromosome 21	94.9341	98.5811
Chromosome 22	128.594	126.32
Chromosome 23	73.622	70.1121

The annotation of *F. taipaiensis* mitogenome were included 34 unique PCGs, comprising 24 core mitochondrial genes essential for oxidative phosphorylation and 10 non-core genes associated with ribosome biogenesis and metabolic regulation (Table 4; Figure 1B). Among the core genes were components of the ATP synthase complex (*atp1*, *atp4*, *atp6*, *atp8*, and *atp9*), NADH dehydrogenase subunits (*nad1*, *nad2*, *nad3*, *nad4*, *nad4L*, *nad5*, *nad6*, *nad7*, and *nad9*), cytochrome c biogenesis genes (*ccmB*, *ccmC*, *ccmFC*, and *ccmFN*), cytochrome c oxidase subunits (*cox1*, *cox2*, and *cox3*), the membrane transporter *mttB*, and regulatory genes *matR* and *cob*. Three genes, *atp9*, *ccmC*, and *matR*, were each present in two copies. The non-core genes included ribosomal protein genes from the large (*rpl5*, *rpl10*, and *rpl16*) and small (*rps2*, *rps3*, *rps12*, *rps13*, *rps14*, and *rps19*) subunits, as well as the tricarboxylic acid cycle gene *sdh4*. In addition, three mitochondrial rRNA genes—*rrn5*, *rrn18*, and *rrn26*—were identified. A total of 12 tRNA genes were detected, including *trnC-GCA*, *trnD-GUC*, *trnE-UUC*, *trnfM-CAU*, *trnI-CAU*, *trnK-UUU*, *trnM-CAU*, *trnN-GUU*, *trnQ-UUG*, *trnT-UGU*, *trnW-CCA*, and *trnY-GUA*. Among these, *trnT-UGU* occurred in two copies and *trnM-CAU* in three copies.

**TABLE 4:** Gene composition in the mitogenome of *F. taipaiensis.*

Group of genes	Name of genes
ATP synthase	*atp1, atp4, atp6, atp8, atp9* (×2)
NADH dehydrogenase	*nad1, nad2, nad3, nad4, nad4L, nad5, nad6, nad7, nad9*
Cytochrome b	*cob*
Cytochrome c biogenesis	*ccmB, ccmC* (×2), *ccmFC, ccmFN*
Cytochrome c oxidase	*cox1, cox2, cox3*
Maturases	*matR* (×2)
Protein transport subunit	*mttB*
Ribosomal protein large subunit	*rpl5, rpl10, rpl16*
Ribosomal protein small subunit	*rps2, rps3, rps12, rps13, rps14, rps19*
Succinate dehydrogenase	*sdh4*
Ribosome RNA	*rrn5, rrn18, rrn26*
Transfer RNA	*trnC-GCA, trnD-GUC, trnE-UUC, trnfM-CAU, trnI-CAU, trnK-UUU, trnM-CAU* (×3), *trnN-GUU, trnQ-UUG, trnT-UGU* (×2)*, trnW-CCA, trnY-GUA*

The number following gene names indicates the number of copies, for example, *trnM-CAU*, had three copies.

### 3.2 Repeat elements and prediction of RNA editing events

**FIGURE 2:**
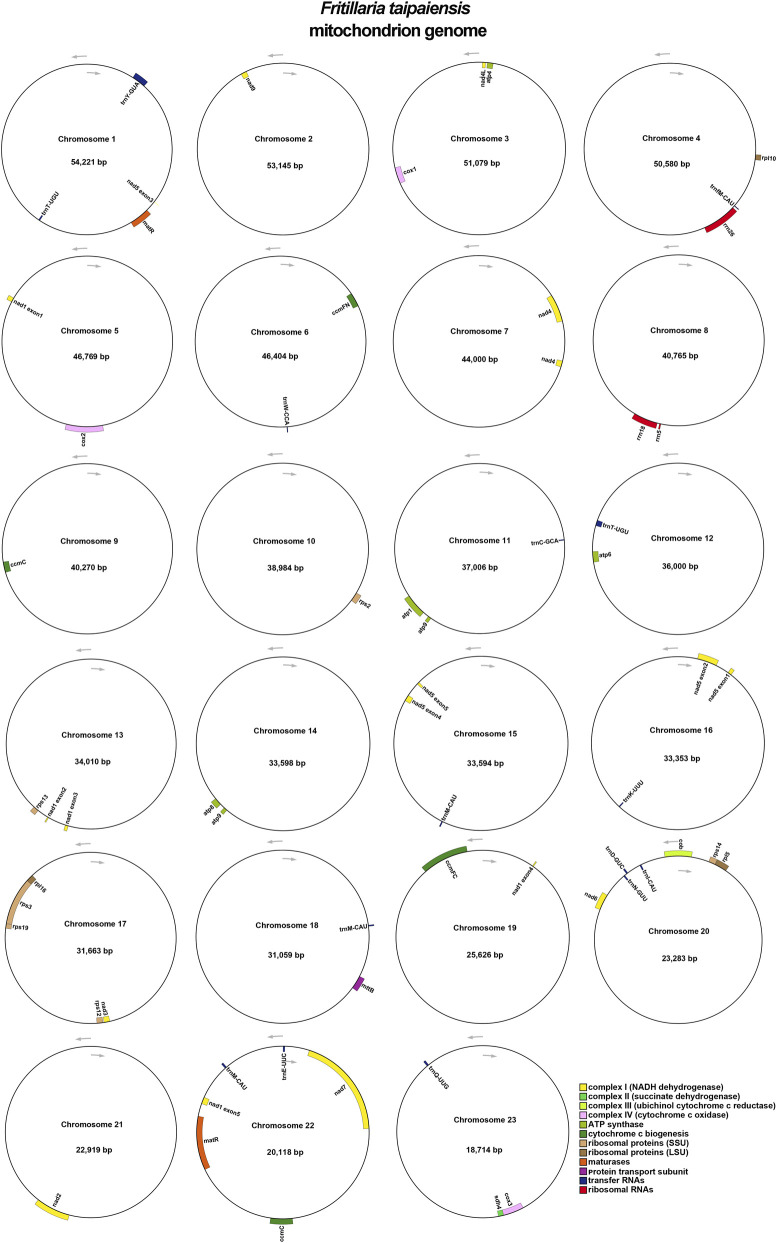
The map of the F. taipaiensis mitogenome. The arrows shown transcriptional direction of the mitogenome. Genes with different functions were depicted using different colors.

In the *F. taipaiensis* mitogenome, a total of 178 simple sequence repeats (SSRs) were identified (Figure 2A), among which tetrameric repeats were the most abundant, comprising 65 SSRs and accounting for 36.52% of the total. The numbers of monomeric, dimeric, and trimeric SSRs were relatively similar, with 32, 48, and 27 occurrences, respectively. In contrast, only five pentameric and a single hexameric repeat were detected, indicating a low prevalence of longer repeat motifs. The cumulative length of all SSRs was 2,137 bp. Additionally, analysis of dispersed repeats revealed 127 sequences, including 59 forward repeats and 68 palindromic repeats (Figure 2B), ranging in length from 30 to 341 bp. No reverse or complementary repeats were identified (Figure 2C). Furthermore, 13 tandem repeats were detected, varying in size from 30 to 72 bp, highlighting the structural complexity and sequence diversity of the *F. taipaiensis* mitogenome.

**FIGURE 3:**
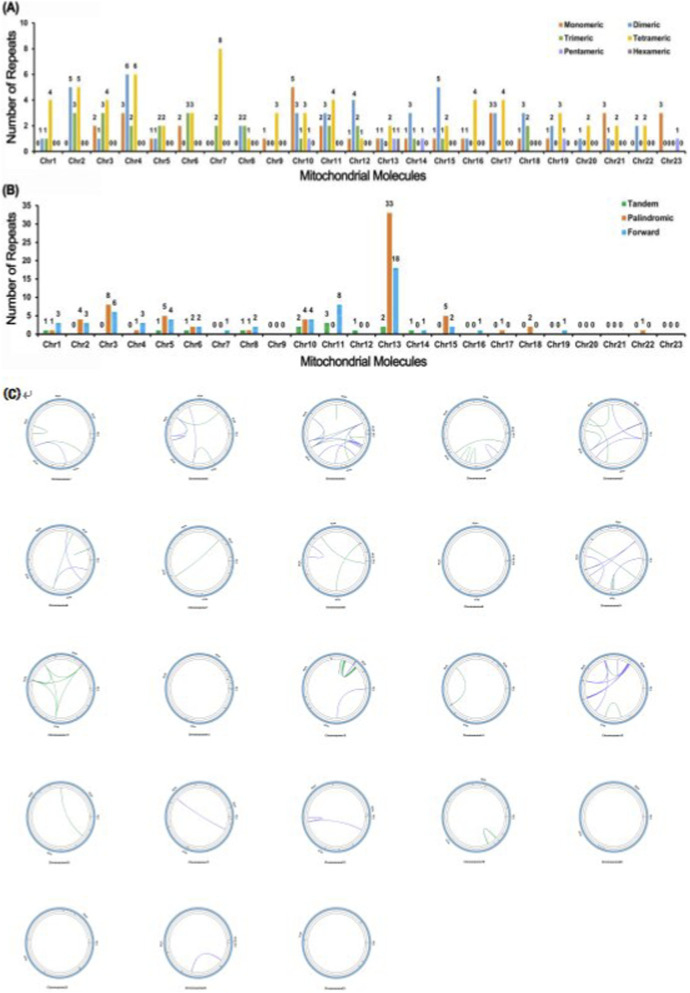
Analysis of repeat elements in the mitogenome of F. taipaiensis. **(A)** Distribution of simple sequence repeats classified by repeat unit length (monomeric, dimeric, trimeric, tetrameric, pentameric, and hexameric) across the 23 chromosomes of *F. taipaiensis*. **(B)** Classification of repeats based on structural types, including tandem, palindromic, forward, reverse, and complementary repeats. **(C)** Chord diagram of repeat sequence analysis for 23 chromosomes of *F. taipaiensis*. **(A)** The horizontal axis represents mitochondrial molecules, the vertical axis represents the number of repeated fragments, the orange legend represents monomeric SSRs, the blue legend represents dimeric SSRs, the green legend represents trimeric SSRs, the yellow legend represents tetrameric SSRs, the purple legend represents pentameric SSRs, and the gray legend represents hexameric SSRs. **(B)** The horizontal axis represents mitochondrial molecules, the vertical axis represents the number of repeated fragments, the green legend represents Tandem repeats, the orange legend represents Palindromic repeats, and the blue legend represents Forward repeats.

RNA editing analysis revealed that editing frequency was strongly gene-dependent, with NADH dehydrogenase genes exhibiting a particularly high frequency of editing events. Across the 34 annotated PCGs of *F. taipaiensis*, a total of 488 RNA editing sites were predicted, all of which involved cytosine-to-uridine (C-to-U) conversions (Figure 3). A detailed distribution of RNA editing events, including the editing frequency per gene, codon position, and edit type, is provided in Supplementary Table 3. Overall, editing frequency varied markedly among genes, with *nad* genes containing the highest numbers of editing sites, and most edits occurring at the first and second codon positions. By contrast, only 6.15% of editing sites (30 times) occurred at the third codon position, where synonymous changes are more likely. Among all genes, *nad4* exhibited the highest number of editing sites (47 times), while most genes had between 5 and 36 editing events. A subset of genes (*atp8*, *nad2*, *rpl10*, *rps2*, *rps19*, and *sdh4*) contained fewer than five editing sites, and no RNA editing site was detected in *rps14*.

Several RNA editing events were predicted to have the potential to influence protein coding capacity. Two major categories of putatively high-impact RNA editing were observed: changes that may alter translation initiation and edits predicted to introduce premature stop codons. RNA editing also contributed to the creation of functional start and stop codons (Table 5). For example, an ACG codon in *nad4L* was edited to AUG, generating a canonical start codon, while a CGA codon in *ccmFC* was edited to UGA, resulting in a stop codon. Additionally, a predicted editing event converting an arginine codon into a stop codon was detected in *ccmFC*, which may lead to premature termination of translation. These observations suggest that RNA editing may play an important role in shaping mitochondrial protein coding sequences in *F. taipaiensis*, although experimental validation (e.g., RNA-seq) will be required to confirm these effects.

**TABLE 5:** RNA editing in the mitogenome of *F. taipaiensis*

Gene	Base position	Amino position	Codon change	Amino change
*atp4*	326	109	ACG->AUG	Thr->Met
*cox2*	443	148	ACG->AUG	Thr->Met
*cox2*	698	233	ACG->AUG	Thr->Met
*nad4L*	2	1	ACG->AUG	Thr->Met
*ccmFC*	1,306	436	CGA->UGA	Arg->End

### 3.3 Intracellular gene transfer

**FIGURE 4:**
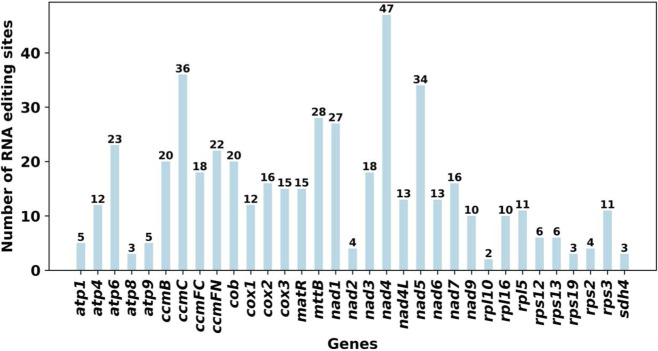
Prediction of RNA editing sites based on the *F. taipaiensis* PCGs. The bar chart illustrates the number of predicted RNA editing sites across various protein-coding genes (PCGs) within the *F. taipaiensis* mitogenome. The x-axis lists the genes, while the y-axis represents the number of RNA editing sites predicted for each gene.

Numerous studies have emphasized the significance of mitochondrial plastid DNA sequences (MTPTs) in improving the accuracy of chloroplast and mitogenome assembly, as well as in elucidating the evolutionary dynamics of organelle genomes. The plastome obtained in this study (GenBank accession: PV819102) exhibited a structure highly consistent with the previously reported genome (MK642356). Pairwise comparison of the two chloroplast genomes revealed a high degree of sequence conservation, with an average nucleotide identity of 99.93%, and no major rearrangements were detected, as illustrated in the dot plot (Supplementary Figure S4). In the *F. taipaiensis* mitogenome, we identified 10 MTPTs that have been transferred into the mitogenome (Table 6). These transferred fragments collectively span 6,788 bp, accounting for approximately 0.80% of the total mitogenome (Figure 4). Among them, four MTPTs exceed 200 bp in length, with MTPT1 being the longest at 3,692 bp. In the Nanopore- and Illumina-based mapping results of the mitogenome, most MTPT regions exhibited pronounced coverage enrichment. In contrast, MTPT2, MTPT3, and MTPT7 displayed relatively uniform and lower coverage. Notably, although these three regions retain sequence homology to plastid-derived fragments, they lack substantial read support from the chloroplast genome.

**TABLE 6:** The homologous DNA fragment in the mitogenome of *F. taipaiensis*.

Number	Identity%	Alignment Length (bp)	Chloroplast genome		Mitochondrial genome	Mitochondrial genome		MTPT annotation
			Start	End		Start	End	
MTPT1	100	3,692	35,655	39,346	Chr1	26,869	30,560	Partial *psaB*; complete *psaA*
MTPT2	86.66	75	47,804	47,731	Chr2	17,812	17,882	Partial *ndhC*
MTPT3	100	41	60,015	60,055	Chr3	14,108	14,148	Partial *petA*
MTPT4	89.56	1928	32,964	31,047	Chr3	43,364	45,253	Partial *psbD*
MTPT5	96.80	94	63,537	63,444	Chr6	35,319	35,412	Complete *trnW-CCA*
MTPT6	84.58	253	119,186	118,938	Chr11	24,384	24,629	Partial *ndhA*; partial *ndhH*
MTPT7	100	30	91,005	91,034	Chr12	27,431	27,460	IGS (*ycf2-trnL-CAA*)
	100	30	142,152	142,123	Chr12	27,431	27,460	IGS *(trnL-CAA-ycf2*)
MTPT8	91	100	95,149	95,050	Chr18	15,659	15,752	Partial *rps7*
	91	100	138,008	138,107	Chr18	15,659	15,752	Partial *rps7*
MTPT9	77.28	449	97,498	97,065	Chr19	10,758	11,151	IGS (*rps12 -trnV-GAC*)
	77.28	449	135,659	136,092	Chr19	10,758	11,151	IGS (*trnV-GAC-rps12*)
MTPT10	91.27	126	105,817	105,692	Chr21	19,347	19,470	Partial *trnR-ACG*
	91.27	126	127,340	127,465	Chr21	19,347	19,470	Partial *trnR-ACG*

Annotation of these homologous sequences revealed the presence of two intact chloroplast-derived genes, *psaA* and *trnW-CCA*, as well as eight partial gene fragments corresponding to *psaB*, *ndhA*, *ndhC*, *ndhH*, *petA*, *psbD*, *rps7*, and *trnR-ACG*. These findings provide insights into historical DNA transfer events between the chloroplast and mitogenome in *F. taipaiensis* and underscore the role of MTPTs in shaping organelle genome structure and function.

### 3.4 Codon usage analysis and collinearity analysis

**FIGURE 5:**
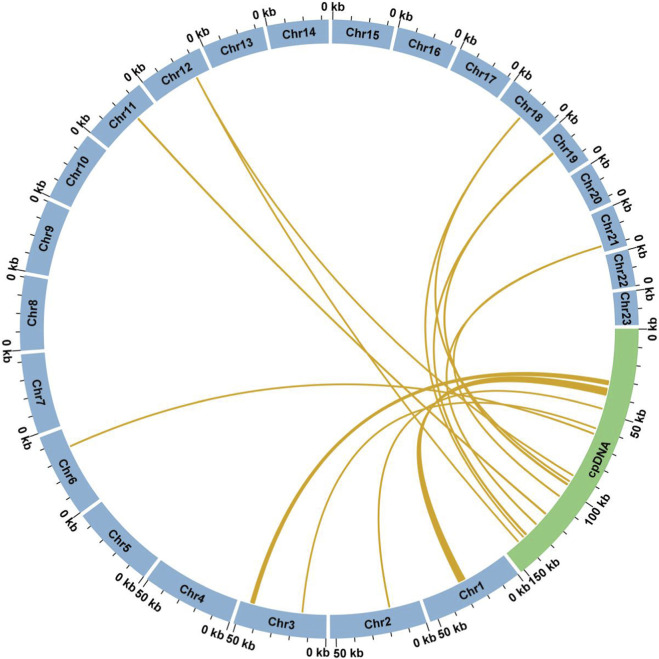
Homologous analysis based on the different organelles. The blue arc represents mitogenome, and the green arc represents chloroplast genome. The homologous fragments are indicated using the yellow lines between blue and green arcs.

**FIGURE 6:**
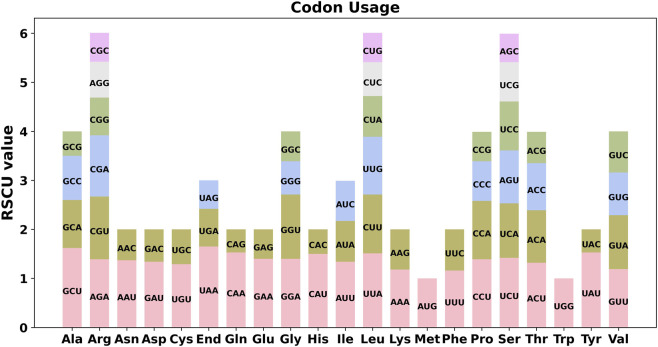
Relative synonymous codon usage (RSCU) in the mitochondrial protein-coding genes of *F. taipaiensis*. Relative synonymous codon usage (RSCU) in the mitochondrial protein-coding genes of *F. taipaiensis*. The figure displays the RSCU values for the 34 unique protein-coding genes in the *F. taipaiensis* mitochondrial genome. The codon usage patterns are represented for 20 amino acids and stop codons (End), showing the preference for certain codons over others. Codons with higher RSCU values indicate a greater frequency of usage relative to other synonymous codons.

Codon usage analysis was conducted on the PCGs of the *F. taipaiensis* mitogenome, with the relative synonymous codon usage (RSCU) values for each amino acid summarized in Table 7. Codons with RSCU values greater than 1 were considered preferentially used. As shown in Figure 5, aside from the start codon AUG (Methionine) and UGG (Tryptophan)—both exhibiting RSCU values of 1—several codons demonstrated clear usage bias. The most frequently used codons included GCU (Alanine, RSCU = 1.62), UAU (Tyrosine, RSCU = 1.53), and CAA (Glutamine, RSCU = 1.53), suggesting notable codon usage preferences across mitochondrial PCGs in *F. taipaiensis*. Across the 34 mitochondrial PCGs of F. taipaiensis, ENC values ranged from 51.74 to 58.96, indicating generally weak codon usage bias, which was also supported by relatively low CAI values (Figures 6A,B). Most genes exhibited GC3s values below 40%, reflecting an AT-rich composition at the third codon position. In the ENC–GC3s plot, most genes were distributed close to or slightly below the expected curve, suggesting that mutation bias plays a predominant role in shaping codon usage patterns, while a few genes (e.g., *matR*, *ccmFC*, and *ccmFN*) may also be influenced by weak selective constraints.

**TABLE 7:** Relative synonymous codon usage for each amino acid in the mitogenome of *F. taipaiensis*.

Amino	Codon 1	Codon 2	Codon 3	Codon 4	Codon 5	Codon 6
	RSCU	RSCU	RSCU	RSCU	RSCU	RSCU
Ala	GCU	GCA	GCC	GCG	​	​
	1.62	0.98	0.9	0.5	​	​
Arg	AGA	CGU	CGA	CGG	AGG	CGC
	1.39	1.28	1.25	0.77	0.73	0.59
Asn	AAU	AAC	​	​	​	​
	1.37	0.63	​	​	​	​
Asp	GAU	GAC	​	​	​	​
	1.34	0.66	​	​	​	​
Cys	UGU	UGC	​	​	​	​
	1.29	0.71	​	​	​	​
End	UAA	UGA	UAG	​	​	​
	1.65	0.77	0.58	​	​	​
Gln	CAA	CAG	​	​	​	​
	1.53	0.47	​	​	​	​
Glu	GAA	GAG	​	​	​	​
	1.4	0.6	​	​	​	​
Gly	GGA	GGU	GGG	GGC	​	​
	1.4	1.31	0.68	0.61	​	​
His	CAU	CAC	​	​	​	​
	1.5	0.5	​	​	​	​
Ile	AUU	AUA	AUC	​	​	​
	1.34	0.83	0.82	​	​	​
Leu	UUA	CUU	UUG	CUA	CUC	CUG
	1.51	1.2	1.18	0.83	0.69	0.6
Lys	AAA	AAG	​	​	​	​
	1.18	0.82	​	​	​	​
Met	AUG	​	​	​	​	​
	1.0	​	​	​	​	​
Phe	UUU	UUC	​	​	​	​
	1.16	0.84	​	​	​	​
Pro	CCU	CCA	CCC	CCG	​	​
	1.39	1.19	0.81	0.6	​	​
Ser	UCU	UCA	AGU	UCC	UCG	AGC
	1.42	1.11	1.08	1.0	0.8	0.58
Thr	ACU	ACA	ACC	ACG	​	​
	1.32	1.07	0.96	0.64	​	​
Trp	UGG	​	​	​	​	​
	1.0	​	​	​	​	​
Tyr	UAU	UAC	​	​	​	​
	1.53	0.47	​	​	​	​
Val	GUU	GUA	GUG	GUC	​	​
	1.19	1.1	0.87	0.84	​	​

**FIGURE 7:**
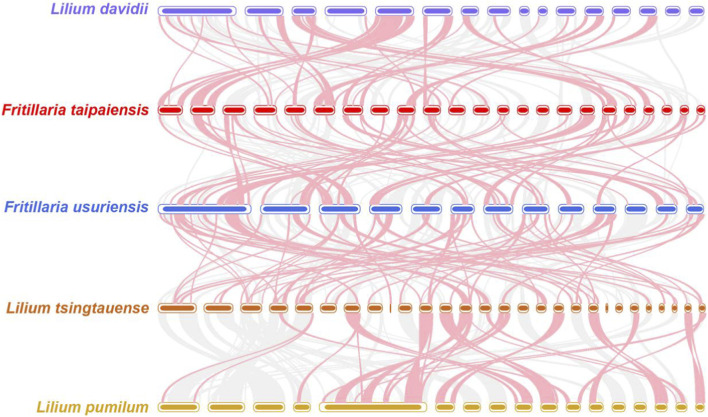
Collinear analysis of five Liliaceae species. The pink arcs indicated inverted regions. The gray arcs indicated better homologous regions. The regions with no colinear blocks are indicated as unique in the species.

To investigate structural variation and evolutionary divergence, a comparative mitogenomic analysis was performed between *F. taipaiensis* and four related species: *F*. *ussuriensis* (OR783162-OR783174), *L*. *davidii* (MT985331-MT985346), *L*. *tsingtauense* (OP973783-OP973810), and *L*. *pumilum* (MT985347-MT985363). Dot-plot analysis, using *F. taipaiensis* as the reference genome (Figure 7), revealed limited conserved syntenic blocks, each less than 12 Kb in length. The presence of extensive large-scale rearrangements across the mitogenomes highlights a high degree of structural plasticity and suggests poor conservation of mitogenomic architecture within these taxa of Liliaceae (Liliales). These findings reflect substantial genomic restructuring events that likely occurred during the evolutionary divergence of these species.

### 3.5 Construction of phylogenetic tree based on the PCGs

**FIGURE 8:**
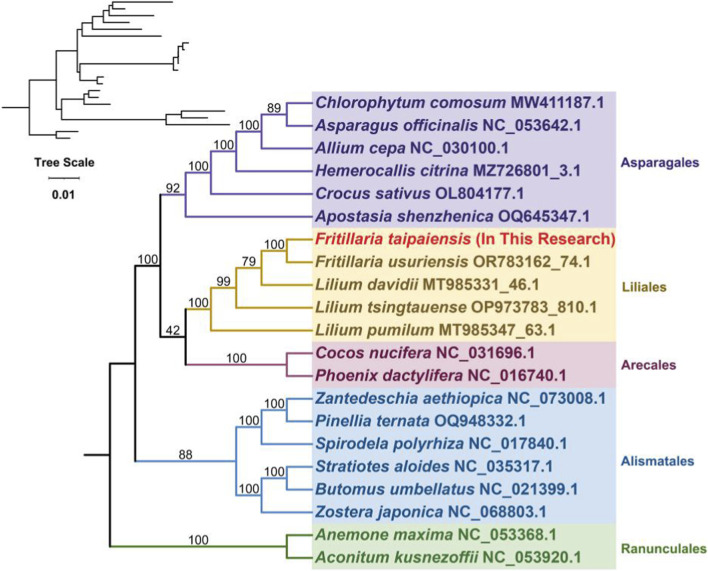
Construction of the maximum likelihood (ML) tree based on the mitogenomes of 21 species. The phylogenetic tree was constructed using the maximum likelihood method based on the shared PCGs (*atp1, atp4, atp6, atp8, atp9, ccmB, ccmFN, ccmFC, ccmC, cox1, cox2, cox3, nad1, nad2, nad3, nad4, nad4L, nad5, nad6, nad7, nad9, matR, mttB,* and* cob*) of 21 species, representing different orders within monocots and one group from Ranunculales. *F. taipaiensis* (highlighted in red) from this study is grouped within the order Asparagales. Bootstrap support values are displayed at the nodes, indicating the confidence levels for the branching pattern.

To resolve the evolutionary relationships of *F. taipaiensis* within Asparagales, we reconstructed a phylogenetic tree based on 24 conserved mitochondrial PCGs shared by all species analyzed (Figure 8). As anticipated, *F. taipaiensis* clustered most closely with *F. ussuriensis*, reflecting their close taxonomic relationship. Additionally, *L. davidii*, *L. pumilum*, and *L. tsingtauense*, all members of the same angiosperm family, formed a well-supported clade with *F. taipaiensis*, consistent with their high degree of gene sequence homology. Species from Arecales, Alismatales, and Asparagales, also clustered according to their recognized taxonomic groupings, thereby supporting the phylogenetic accuracy of the organelle-based analysis.

## 4 Discussion

### 4.1 Structure of the mitogenomes of *Fritillaria* genus

Mitochondria represent pivotal organelles within eukaryotic cells, serving as the principal energy suppliers for diverse cellular physiological processes. Plant mitogenomes exhibit a more intricate architectural complexity compared to their animal counterparts, characterized by substantial size fluctuations, extensive sequence collinearity, abundant repetitive elements, and highly conserved coding sequences (Møller et al., 2021). An increasing number of studies have reported plant mitogenome sequences, advancing our understanding of their genetic architecture. While plant mitogenomes are often depicted as single circular maps, several studies have revealed that they can also exist as multipartite, multi-circular structures (Mower et al., 2012; Sloan et al., 2012; Kozik et al., 2019). Similarly, it predominantly manifests as a dynamic and complex assemblage of multiple circular DNA molecules, interspersed with smaller circular DNA structures, forming a sophisticated genomic network (Taanman, 1999; Wynn and Christensen, 2019; Jackman et al., 2020). Before this study, only the mitogenome of *F. ussuriensis* had been available (Xie et al., 2024) (OR783162–OR783174), limiting comparative and evolutionary analyses within *Fritillaria*. In this research, we assembled the mitogenome of *F. taipaiensis* (PV819105–PV819127), which spans 847,160 bp—longer than the 737,569 bp mitogenome of *F. ussuriensis*. The GC content of *F. taipaiensis* was 44.98%, very similar to that of *F. ussuriensis* (45.41%), indicating similar GC proportion between the two mitogenomes. The mitogenome of *F. taipaiensis* exhibits a multi-circular architecture, consisting of 23 circular chromosomes with sizes ranging relatively uniformly from 18,714 bp to 54,221 bp. In contrast to *F. taipaiensis*, the mitogenome of *F. ussuriensis* comprises 13 circular chromosomes, including one exceptionally large chromosome of 154,202 bp, while the remaining chromosomes are predominantly around 50,000 bp in size.

Although the two species exhibit high morphological similarity, their growth environments differ remarkably: *F. ussuriensis* is distributed in northeastern China, where it adapts to a cold and arid climate, whereas *F. taipaiensis* grows in southwestern China, characterized by a warm and humid climate. As mitochondria are core organelles involved in plant energy metabolism, they are likely to play important roles in plant–environment interactions. Therefore, investigating the mitogenomes of these two species may provide insights into the potential relationship between mitochondrial variation and environmental adaptation, although direct causal links remain to be clarified.

### 4.2 Sequence characteristics of the *F. taipaiensis* mitogenome

Eukaryotic genomes contain 64 codons, and the frequency at which these codons are used shows considerable variation among species and taxa. This codon usage bias is thought to result from long-term evolutionary processes acting within cells. It reflects a dynamic balance among mutational biases, translational efficiency, and tRNA abundance, which together shape codon preference across eukaryotic lineages (Zhou and Li, 2009; Sloan et al., 2018). In *F. taipaiensis*, 31 codons showed RSCU values greater than 1.00, of which 87.1% ended with A or U at the third codon position (12 A-ended and 15 U-ended codons). A similar bias toward A/U-ending codons has also been reported in *F. ussuriensis* (>75%) (Xie et al., 2024), *Camellia sinensis* var. duntsa (>63%) (Li et al., 2023), *Cucumis sativus* (>80%) (Niu et al., 2021), and *Stemona tuberosa* (>90%) (Xu et al., 2025). Overall, the strong preference for A/U-ending codons observed in both *F. taipaiensis* and *F. ussuriensis* mitogenomes is consistent with the generally AT-rich nature of plant mitogenomes. Although the two species share broadly similar codon preference profiles, the relative enrichment of codons such as GCU, UAU, and CAA in *F. taipaiensis* suggests subtle lineage-specific differences in codon selection patterns. This pattern is also reflected in the GC distribution across codon positions in *F. taipaiensis*, together with generally high ENC values, indicating weak overall codon usage bias. These differences may reflect species-specific variation in mutational pressure or relaxed translational selection acting on mitochondrial genes, rather than major functional divergence between the two mitogenomes.

Repeated sequences exhibit extensive distribution in mitogenomes, taxonomically categorized into two primary classes: tandem repeats and interspersed repeats. These repetitive elements play a critical role in mediating intermolecular recombination events within mitogenomes (Jiang et al., 2023). Specifically, the largest repeat units within a species-typically exceeding 1 Kb in angiosperms-have been observed to undergo constitutive recombination, a process that drives genomic isomerization (Knoll et al., 2014; Kozik et al., 2019). This recombination-mediated isomerization contributes to the structural plasticity of plant mitogenomes, facilitating both genetic diversity and functional regulation across evolutionary timescales. However, in *F. taipaiensis*, no large repeats were found to be shared among the circular chromosomes, and no clear evidence of inter-chromosomal homologous recombination was detected. Simple sequence repeat (SSR) analysis identified 180 SSRs in *F. taipaiensis*, all shorter than 341 bp, which is comparable to *F. ussuriensis* (192 SSRs, up to 412 bp). Although the overall abundance and size distribution were similar between the two species, differences were observed in the composition and type of SSR motifs, indicating lineage-specific accumulation patterns.

### 4.3 Inter-organellar DNA transfer and homologous fragments

Empirical studies have established that MTPTs serve as crucial genomic markers for deciphering the evolutionary trajectories of chloroplast and mitochondrial genomes (Li S. et al., 2024; Wang et al., 2025). These inter compartmental DNA transfers not only facilitate the structural integration of organelle genomes but also act as evolutionary drivers, shaping the genetic architecture through processes such as homologous recombination and sequence divergence (Wang et al., 2018).

In this study, MTPT regions also correspond to local long-read coverage peaks observed along several mitochondrial chromosomes (Supplementary Figures S1–S3), reflecting their sequence composition and copy number characteristics rather than structural connections between contigs. We observed heterogeneous read coverage among different MTPT regions, with most MTPTs showing pronounced coverage enrichment, whereas MTPT2, MTPT3, and MTPT7 exhibited relatively uniform coverage despite retaining sequence homology to plastid-derived fragments. This difference likely reflects variation in the ability of individual MTPTs to recruit chloroplast-derived reads during mapping. This pattern suggests that the elevated coverage observed in other MTPTs is primarily attributable to the recruitment of chloroplast-derived reads rather than to assembly artifacts or the accumulation of overlapping partial long-read alignments.

Plastid-to-mitochondrion DNA transfer has also been documented in other *Fritillaria* species, where chloroplast-encoded tRNA genes have been experimentally confirmed to be expressed in the mitochondria and to participate in amino-acid transport. Such findings highlight the evolutionary significance of intergenomic DNA trafficking in shaping plant mitogenomes (Kubo and Newton, 2008; Gualberto et al., 2014; Chevigny et al., 2020; Qian et al., 2021). In *F. taipaiensis*, only *psaA* and *trnW-CCA* were found to be completely transferred into the mitogenome, the total length of MTPTs was 6,788 bp, accounting for only 0.8% of the mitogenome. By contrast, in *F. ussuriensis*, several plastid genes were fully transferred, including *rps7, rps12, trnQ-UUG, trnC-GCA, trnW-CCA, rrn5, trnfM-CAU,* and *trnH-GUG*. For example, ten MTPTs totaling 8,954 bp were detected between the mitochondrial and chloroplast genomes of *F. ussuriensis*, accounting for 1.21% of the mitogenome. These results indicate that MTPTs represent a conserved but structurally limited component of the *Fritillaria* mitogenome, contributing to mitogenome evolution and functional diversification without dominating overall genome architecture.

Analysis of collinearity among the five species indicated considerable divergence in mitochondrial genome structure among closely related species of the same genus. The phylogenetic analysis based on 24 conserved mitochondrial PCGs confirms that *F. taipaiensis* occupies a well-defined position within the Liliaceae, clustering closely with *F. ussuriensis* and forming a well-supported clade with other related species in the family, reflecting its close evolutionary relationship within this lineage. Not surprisingly the results reveal well-defined taxonomic relationships within Liliales that are largely consistent with the APG IV system (Chase et al., 2016).

### 4.4 Functional implications of RNA editing in plant mitochondria

RNA editing represents a pervasive post-transcriptional modification in plant mitogenomes, driving substantial diversification of gene sequences. Previous studies have suggested that such edits can, in some cases, influence translation initiation or protein maturation in organelle genome (Sun et al., 2016). To date, cytidine-to-uridine (C-to-U) conversions has been reported as the most common type of RNA editing in plant mitochondria (Wu and Chaw, 2022). A total of 597 RNA editing sites were predicted in sweet potato mitochondria, with *rps3* gene containing the highest number of editing events (54 edits) (Chase et al., 2016). Similarly, 457 RNA editing events were identified in *Rhopalocnemis phalloides* (Yu et al., 2022), 421 RNA editing events in *Acer truncatum* Bunge (Ma et al., 2022) and 479 RNA editing events in *Gossypium raimondii* (Bi et al., 2016). Notably, the editing events reported ih these species were dominated by C-to-U type. A notable observation is the absence of editing sites in the *rps14* gene, which contrasts with the widespread modification patterns seen in other mitochondrial transcripts. Both *F. taipaiensis* and *F. ussuriensis* exhibit extensive C-to-U RNA editing in their mitogenomes (Xie et al., 2024), with editing sites concentrated in NADH dehydrogenase genes and *nad4* showing the highest editing frequency in both species. Most edits occur at the first and second codon positions, indicating a major role in protein recoding rather than synonymous change. In both species, RNA editing also contributes to the formation of functional start and stop codons, particularly in *nad4L* and *ccmFC*. These similarities suggest a conserved role of RNA editing in maintaining mitochondrial gene function in *Fritillaria*, although variation in editing numbers among genes and species indicates lineage-specific differences in editing intensity. However, the observed differences between *F. taipaiensis* and *F. ussuriensis* should be interpreted cautiously, and further functional studies are required to determine whether environmental conditions, such as the relatively warm and humid climate of *F. taipaiensis*, play a role in shaping these gene variation patterns.

## 5 Conclusion

Our study provides the first comprehensive assembly and annotation of the *F. taipaiensis* mitogenome, which consists of 23 circular chromosomes (847,160 bp in total) and 49 genes, revealing its multi-chromosomal structure, repeat content, and RNA editing landscape. These findings not only expand the genomic resources available for *Fritillaria* but also provide valuable insights into the evolution, structural diversity, and functional dynamics of plant mitogenomes. The assembled mitogenome will serve as a foundational reference for future studies on mitochondrial biology, evolutionary relationships, and conservation genetics within the Liliaceae family.

## Sample collection

Compliance with ethical standards Freshly collected, cultivated specimens of *F taipaiensis* were collected from the Hua’e Mountain National Nature Reserve in Dazhou City, Sichuan Province, China (E 108°00′–108°27′, N 31°55′–31°12′). The collection was carried out in accordance with local legislation, and no specific permissions were required for collecting cultivated plant materials within the reserve under current regulations. All samples were taxonomically identified by Prof. Hao Zhang (West China School of Pharmacy, Sichuan University), and voucher specimens have been deposited at the Dazhou Academy of Agricultural Sciences.

Experimental research and plant material collection complied with all relevant institutional, national, and international guidelines.

## Data Availability

The assembled mitochondrial and chloroplast genomes have been deposited in GenBank (NCBI, https://www.ncbi.nlm.nih.gov/) under accession numbers: PV819105–PV819127 and PV819102, respectively. The raw sequencing data are available in GenBank under BioProject: PRJNA1280719, BioSample: SAMN49530828, and SRA accession numbers: SRR34102565 (Illunima)–SRR34102566 (Nanopore).
