# Supplementary material for: Structure and multipartite genome architecture of the mitochondrial genome in the endangered medicinal plant Fritillaria taipaiensis P. Y. Li
Source: Front Syst Biol. 2026 Apr 22;6:1708877. doi: 10.3389/fsysb.2026.1708877 (PMC13143598; doi:10.3389/fsysb.2026.1708877)
Supplement: Supplementary file 2 [file Table3.docx]

**Supplementary Table 3. Prediction of RNA editing in the mitogenome of *F. taipaiensis***

| gene | Base position | Amino position | Codon change | Amino change | Left motifs | Edited site | Right motifs | Probability |
| --- | --- | --- | --- | --- | --- | --- | --- | --- |
| atp1 | 971 | 324 | UCG->UUG | Ser->Leu | CCAGACAGGTGCAGGTAGCT | C | GACTGCCTTACCCGTCATTG | 1 |
| atp1 | 1098 | 366 | CCC->CCU | Pro->Pro | TTTTATCGCGGAATTAGACC | C | GCTATTAACGTTGGCTTATC | 0.958 |
| atp1 | 1415 | 472 | CCA->CUA | Pro->Leu | TCAATATGAGAGAGCCATTC | C | AAGTGGTATAGATCCAGAAT | 0.998 |
| atp1 | 1490 | 497 | CCU->CUU | Pro->Leu | TAACGAAAGAAAGATGGAAC | C | TGATGCTTCTTTAAAAGAAA | 0.995 |
| atp1 | 1499 | 500 | UCU->UUU | Ser->Phe | AAAGATGGAACCTGATGCTT | C | TTTAAAAGAAAGCGCTTTGC | 0.988 |
| atp4 | 56 | 19 | CCA->CUA | Pro->Leu | GATGCTAGTTGCTGCTATTC | C | ATCTATTTGTGCATCAAGTT | 1 |
| atp4 | 59 | 20 | UCU->UUU | Ser->Phe | GCTAGTTGCTGCTATTCCAT | C | TATTTGTGCATCAAGTTCGA | 0.984 |
| atp4 | 71 | 24 | UCA->UUA | Ser->Leu | TATTCCATCTATTTGTGCAT | C | AAGTTCGAAGAAGATCTCAA | 0.995 |
| atp4 | 89 | 30 | UCA->UUA | Ser->Leu | ATCAAGTTCGAAGAAGATCT | C | AATCTATAATGAAGAAATGA | 1 |
| atp4 | 118 | 40 | CGU->UGU | Arg->Cys | ATGAAGAAATGATAGTAGCT | C | GTTGTTTTATAGGCTTTCTC | 0.984 |
| atp4 | 215 | 72 | UCG->UUG | Ser->Leu | CGAGTCTATTCAGGAAGAAT | C | GCAGCAATTCTCCAATCCTA | 0.995 |
| atp4 | 227 | 76 | UCC->UUC | Ser->Phe | GGAAGAATCGCAGCAATTCT | C | CAATCCTAACGAAGTCATTC | 1 |
| atp4 | 248 | 83 | CCU->CUU | Pro->Leu | CAATCCTAACGAAGTCATTC | C | TCCGGAATCCAATCAACAAC | 0.994 |
| atp4 | 250 | 84 | CCG->UCG | Pro->Ser | ATCCTAACGAAGTCATTCCT | C | CGGAATCCAATCAACAACAA | 0.912 |
| atp4 | 326 | 109 | ACG->AUG | Thr->Met | CGTAGTAGAATCATTACCAA | C | GGCACGCTGTGCGCCTAAGT | 0.948 |
| atp4 | 395 | 132 | CCA->CUA | Pro->Leu | CCGAAACCTAAATGTCAAGC | C | AGCAACACTTCTAAATGCCA | 0.997 |
| atp4 | 416 | 139 | ACU->AUU | Thr->Ile | AGCAACACTTCTAAATGCCA | C | TTCTTCCCGTCGCATCCGTC | 0.986 |
| atp6 | 40 | 14 | CCA->UCA | Pro->Ser | TTTATTTATCATTCACAAAT | C | CATCCTTGTCTATGCTCCTC | 0.996 |
| atp6 | 50 | 17 | UCU->UUU | Ser->Phe | ATTCACAAATCCATCCTTGT | C | TATGCTCCTCACTCTCGGTT | 0.998 |
| atp6 | 119 | 40 | UCA->UUA | Ser->Leu | GAAAAAGGGAGGGGGAAAGT | C | AGTGCCAAATGCTTGGCAAT | 1 |
| atp6 | 157 | 53 | CAU->UAU | His->Tyr | AATCCTTGGTAGAGCTTATT | C | ATGATTTCGTGCCGAACCCG | 0.999 |
| atp6 | 170 | 57 | CCG->CUG | Pro->Leu | GCTTATTCATGATTTCGTGC | C | GAACCCGGTAAACGAACAAA | 0.998 |
| atp6 | 176 | 59 | CCG->CUG | Pro->Leu | TCATGATTTCGTGCCGAACC | C | GGTAAACGAACAAATAGGCG | 0.993 |
| atp6 | 227 | 76 | UCC->UUC | Ser->Phe | AAATGTTAAACAAAAGTTTT | C | CCCTCGCATCTCGGTCACTT | 0.995 |
| atp6 | 232 | 78 | CGC->UGC | Arg->Cys | TTAAACAAAAGTTTTCCCCT | C | GCATCTCGGTCACTTTTACT | 0.999 |
| atp6 | 239 | 80 | UCG->UUG | Ser->Leu | AAAGTTTTCCCCTCGCATCT | C | GGTCACTTTTACTTTATCGT | 0.943 |
| atp6 | 257 | 86 | UCG->UUG | Ser->Leu | CTCGGTCACTTTTACTTTAT | C | GTTATTTCGTAATCTGCAGG | 0.907 |
| atp6 | 265 | 89 | CGU->UGU | Arg->Cys | CTTTTACTTTATCGTTATTT | C | GTAATCTGCAGGGTATGATA | 1 |
| atp6 | 404 | 135 | UCA->UUA | Ser->Leu | GCTTCATTTTTTTAGCTTCT | C | ATTACCCGCAGGAGTCCCAC | 1 |
| atp6 | 463 | 155 | CCU->UCU | Pro->Ser | TAGTACTCCTTGAGCTAATC | C | CTCATTGTTTTCGTGCATTA | 0.999 |
| atp6 | 466 | 156 | CAU->UAU | His->Tyr | TACTCCTTGAGCTAATCCCT | C | ATTGTTTTCGTGCATTAAGC | 1 |
| atp6 | 488 | 163 | UCA->UUA | Ser->Leu | TTGTTTTCGTGCATTAAGCT | C | AGGAATACGTTTATTTGCTA | 1 |
| atp6 | 530 | 177 | UCA->UUA | Ser->Leu | TATGATGGCCGGTCATAGTT | C | AGTAAAGATTTTAAGTGGGT | 1 |
| atp6 | 551 | 184 | UCU->UUU | Ser->Phe | AGTAAAGATTTTAAGTGGGT | C | TGCTTGGACTATGCTATTTA | 1 |
| atp6 | 638 | 213 | UCG->UUG | Ser->Leu | AGTTCTAGCATTAACCGGTT | C | GGAATTAGGTGTTGCTATAT | 0.963 |
| atp6 | 659 | 220 | UCA->UUA | Ser->Leu | GGAATTAGGTGTTGCTATAT | C | ACAAGCTCATGTTTCTACGA | 0.998 |
| atp6 | 667 | 223 | CAU->UAU | His->Tyr | GTGTTGCTATATCACAAGCT | C | ATGTTTCTACGATCTCAATC | 0.983 |
| atp6 | 674 | 225 | UCU->UUU | Ser->Phe | TATATCACAAGCTCATGTTT | C | TACGATCTCAATCTGTATTT | 0.997 |
| atp6 | 683 | 228 | UCA->UUA | Ser->Leu | AGCTCATGTTTCTACGATCT | C | AATCTGTATTTACTTGAATG | 1 |
| atp6 | 710 | 237 | ACA->AUA | Thr->Ile | TATTTACTTGAATGATGCTA | C | AAATCTCCATCAAAATAAAG | 0.999 |
| atp8 | 30 | 10 | UUC->UUU | Phe->Phe | CTGGATAAATTTACTTATTT | C | ACACAATTCTTCTGGTCATG | 0.965 |
| atp8 | 47 | 16 | UCA->UUA | Ser->Leu | TTTCACACAATTCTTCTGGT | C | ATGCCTTCTCTTCTTTACTC | 0.999 |
| atp8 | 443 | 148 | CCA->CUA | Pro->Leu | CATAATGCTCATCCATGTTC | C | ACACGGCCAAGGAAGCATCG | 0.993 |
| atp9 | 20 | 7 | UCA->UUA | Ser->Leu | GATGTTAGAGGGAGCAAAAT | C | AATAGGTGCGGGAGCTGCTA | 0.989 |
| atp9 | 50 | 17 | UCA->UUA | Ser->Leu | GGGAGCTGCTACAATTGCTT | C | AGCGGGGGCTGCTGTCGGTA | 0.999 |
| atp9 | 81 | 27 | GUC->GUU | Val->Val | GCTGTCGGTATTGGAAATGT | C | CTCAGTTCTTTGATTCATTC | 0.956 |
| atp9 | 82 | 28 | CUC->UUC | Leu->Phe | CTGTCGGTATTGGAAATGTC | C | TCAGTTCTTTGATTCATTCC | 1 |
| atp9 | 134 | 45 | UCA->UUA | Ser->Leu | TCCATCATTGGCTAAACAAT | C | ATTTGGTTATGCAATTTTGG | 0.999 |
| ccmB | 28 | 10 | CAU->UAU | His->Tyr | GACTCTTTCTTGAACTATAT | C | ATAAACAGATCCTCTCCTCT | 0.986 |
| ccmB | 80 | 27 | UCG->UUG | Ser->Leu | GAGTTTTTCTCTATTCCTCT | C | GTATATCGTCGTAACGCCCT | 0.982 |
| ccmB | 87 | 29 | AUC->AUU | Ile->Ile | TCTCTATTCCTCTCGTATAT | C | GTCGTAACGCCCTTAATGCT | 0.982 |
| ccmB | 128 | 43 | UCA->UUA | Ser->Leu | AGGTTTTGAAAAAGACTTTT | C | ATGTCATTCCCATTTAGGTC | 0.998 |
| ccmB | 137 | 46 | UCC->UUC | Ser->Phe | AAAAGACTTTTCATGTCATT | C | CCATTTAGGTCCGATTCGGA | 0.988 |
| ccmB | 148 | 50 | CCG->UUG | Pro->Leu | CATGTCATTCCCATTTAGGT | C | CGATTCGGATCCCTCCGTTG | 0.989 |
| ccmB | 149 | 50 | CCG->UUG | Pro->Leu | ATGTCATTCCCATTTAGGTC | C | GATTCGGATCCCTCCGTTGT | 0.996 |
| ccmB | 154 | 52 | CGG->UGG | Arg->Trp | ATTCCCATTTAGGTCCGATT | C | GGATCCCTCCGTTGTTTCCT | 0.978 |
| ccmB | 160 | 54 | CCU->UCU | Pro->Ser | ATTTAGGTCCGATTCGGATC | C | CTCCGTTGTTTCCTTTTCCT | 0.992 |
| ccmB | 164 | 55 | CCG->CUG | Pro->Leu | AGGTCCGATTCGGATCCCTC | C | GTTGTTTCCTTTTCCTTCCG | 0.99 |
| ccmB | 172 | 58 | CCU->UCU | Pro->Ser | TTCGGATCCCTCCGTTGTTT | C | CTTTTCCTTCCGCACCTTTT | 0.99 |
| ccmB | 179 | 60 | CCU->CUU | Pro->Leu | CCCTCCGTTGTTTCCTTTTC | C | TTCCGCACCTTTTCCTCGAA | 0.97 |
| ccmB | 193 | 65 | CCU->UUU | Pro->Phe | CTTTTCCTTCCGCACCTTTT | C | CTCGAAATGAGAAAGAAGAT | 0.984 |
| ccmB | 194 | 65 | CCU->UUU | Pro->Phe | TTTTCCTTCCGCACCTTTTC | C | TCGAAATGAGAAAGAAGATG | 0.999 |
| ccmB | 286 | 96 | CGG->UGG | Arg->Trp | TTCTACAATTGGTGGGTCAC | C | GGGTTATTCAAATAAGTCGT | 0.976 |
| ccmB | 304 | 102 | CGU->UGU | Arg->Cys | ACCGGGTTATTCAAATAAGT | C | GTGTTTTCTGTGGTTTTCCC | 0.996 |
| ccmB | 338 | 113 | CCG->CUG | Pro->Leu | TTTTCCCATGTTACAACTTC | C | GTACCAATTCGGTCGATCCG | 0.997 |
| ccmB | 367 | 123 | CGG->UGG | Arg->Trp | TCGGTCGATCCGGAATGGAT | C | GGTTAAACATTCCATTAGGG | 0.979 |
| ccmB | 379 | 127 | CCA->UUA | Pro->Leu | GAATGGATCGGTTAAACATT | C | CATTAGGGAGCCTGGTCTTG | 0.919 |
| ccmB | 380 | 127 | CCA->UUA | Pro->Leu | AATGGATCGGTTAAACATTC | C | ATTAGGGAGCCTGGTCTTGA | 0.999 |
| ccmC | 5 | 2 | UCC->UUC | Ser->Phe | AGGACAAAATTATCACATGT | C | CCTTTCGTTATTACAACCTT | 0.973 |
| ccmC | 76 | 26 | CGG->UGG | Arg->Trp | CGCAAATTATCATTGGATCT | C | GGTTGTTCTTAACAGCGATG | 0.999 |
| ccmC | 103 | 35 | CAU->UAU | His->Tyr | TCTTAACAGCGATGGCTATT | C | ATTTAAGTCTTCGGGTAGCA | 0.999 |
| ccmC | 115 | 39 | CGG->UGG | Arg->Trp | TGGCTATTCATTTAAGTCTT | C | GGGTAGCACCACCAGATCTT | 0.989 |
| ccmC | 133 | 45 | CUU->UUU | Leu->Phe | TTCGGGTAGCACCACCAGAT | C | TTCAACAAGGTGGAAATTCT | 0.957 |
| ccmC | 161 | 54 | CCG->CUG | Pro->Leu | AGGTGGAAATTCTCGTATTC | C | GTATGTACATGTTCCTGCGG | 1 |
| ccmC | 179 | 60 | GCG->GUG | Ala->Val | TCCGTATGTACATGTTCCTG | C | GGCTCGGATGAGTATAGTAA | 0.998 |
| ccmC | 184 | 62 | CGG->UGG | Arg->Trp | ATGTACATGTTCCTGCGGCT | C | GGATGAGTATAGTAATTTAT | 0.996 |
| ccmC | 236 | 79 | CCA->CUA | Pro->Leu | TATAAACAGTTTATTGTTCC | C | ATTAACAAAACATCCCCTTT | 0.986 |
| ccmC | 281 | 94 | ACA->AUA | Thr->Ile | TCGCTCTTCCGGAACCGGTA | C | AGAAATTGGTGCTTTTTCTA | 0.903 |
| ccmC | 299 | 100 | UCU->UUU | Ser->Phe | TACAGAAATTGGTGCTTTTT | C | TACTTTGTTTACCTTAGTTA | 0.97 |
| ccmC | 331 | 111 | CGG->UGG | Arg->Trp | CCTTAGTTACTGGGGGGCTT | C | GGGGAAGGCCTATGTGGGGT | 0.986 |
| ccmC | 358 | 120 | CGG->UGG | Arg->Trp | GGCCTATGTGGGGTACCTTT | C | GGGTGTGGGATGCTCGTTTA | 0.999 |
| ccmC | 400 | 134 | CUU->UUU | Leu->Phe | CTTCTGTATTAATCTTGTTC | C | TTATTTACCTGGGTGCACTG | 0.996 |
| ccmC | 421 | 141 | CGU->UGU | Arg->Cys | TTATTTACCTGGGTGCACTG | C | GTTTTCAAAAGCTTTCTGTC | 0.999 |
| ccmC | 446 | 149 | CCG->CUG | Pro->Leu | TCAAAAGCTTTCTGTCGAAC | C | GGCTCATATTTCAATCCGTG | 0.995 |
| ccmC | 451 | 151 | CAU->UAU | His->Tyr | AGCTTTCTGTCGAACCGGCT | C | ATATTTCAATCCGTGCTGGA | 0.999 |
| ccmC | 458 | 153 | UCA->UUA | Ser->Leu | TGTCGAACCGGCTCATATTT | C | AATCCGTGCTGGACCGATCG | 0.913 |
| ccmC | 463 | 155 | CGU->UGU | Arg->Cys | AACCGGCTCATATTTCAATC | C | GTGCTGGACCGATCGATATA | 0.99 |
| ccmC | 467 | 156 | GCU->GUU | Ala->Val | GGCTCATATTTCAATCCGTG | C | TGGACCGATCGATATACCAA | 0.959 |
| ccmC | 473 | 158 | CCG->CUG | Pro->Leu | TATTTCAATCCGTGCTGGAC | C | GATCGATATACCAATAATCA | 0.995 |
| ccmC | 497 | 166 | UCU->UUU | Ser->Phe | CGATATACCAATAATCAAGT | C | TCCAGTCAACTGGTGGAATA | 0.999 |
| ccmC | 499 | 167 | CCA->UCA | Pro->Ser | ATATACCAATAATCAAGTCT | C | CAGTCAACTGGTGGAATACA | 0.997 |
| ccmC | 521 | 174 | UCG->UUG | Ser->Leu | AGTCAACTGGTGGAATACAT | C | GCATCAACCTGGGAGCATTA | 0.999 |
| ccmC | 548 | 183 | UCU->UUU | Ser->Phe | ACCTGGGAGCATTAGCCGAT | C | TGGTACATCCATACATGTTC | 0.943 |
| ccmC | 568 | 190 | CCU->UCU | Pro->Ser | CTGGTACATCCATACATGTT | C | CTATGCCCATTCCAATCTTG | 0.996 |
| ccmC | 575 | 192 | CCC->CUC | Pro->Leu | ATCCATACATGTTCCTATGC | C | CATTCCAATCTTGTCCAACT | 0.993 |
| ccmC | 591 | 197 | UCC->UCU | Ser->Ser | ATGCCCATTCCAATCTTGTC | C | AACTTTGCTAACTCCCCCTT | 0.987 |
| ccmC | 605 | 202 | UCC->UUC | Ser->Phe | CTTGTCCAACTTTGCTAACT | C | CCCCTTCTCAACCTCTATCT | 0.998 |
| ccmC | 608 | 203 | CCC->CUC | Pro->Leu | GTCCAACTTTGCTAACTCCC | C | CTTCTCAACCTCTATCTTCT | 0.994 |
| ccmC | 614 | 205 | UCA->UUA | Ser->Leu | CTTTGCTAACTCCCCCTTCT | C | AACCTCTATCTTCTTCGTTC | 0.995 |
| ccmC | 630 | 210 | UUC->UUU | Phe->Phe | TTCTCAACCTCTATCTTCTT | C | GTTCTGGAAACACGTCTTCC | 0.949 |
| ccmC | 650 | 217 | CCU->CUU | Pro->Leu | CGTTCTGGAAACACGTCTTC | C | TATTCCATCTTTTCCCGAAT | 0.982 |
| ccmC | 656 | 219 | CCA->CUA | Pro->Leu | GGAAACACGTCTTCCTATTC | C | ATCTTTTCCCGAATCTCCCT | 0.989 |
| ccmC | 665 | 222 | CCC->CUC | Pro->Leu | TCTTCCTATTCCATCTTTTC | C | CGAATCTCCCTTAACTGAAG | 0.963 |
| ccmC | 673 | 225 | CCC->UCC | Pro->Ser | TTCCATCTTTTCCCGAATCT | C | CCTTAACTGAAGAAATAGAA | 0.97 |
| ccmFC | 38 | 13 | UCC->UUC | Ser->Phe | CTTCTTCTTTTTCATTACTT | C | CATGGTCGTGCCTCGTGGCA | 0.999 |
| ccmFC | 50 | 17 | CCU->CUU | Pro->Leu | CATTACTTCCATGGTCGTGC | C | TCGTGGCACGGCAGCACCCG | 1 |
| ccmFC | 52 | 18 | CGU->UGU | Arg->Cys | TTACTTCCATGGTCGTGCCT | C | GTGGCACGGCAGCACCCGTA | 0.984 |
| ccmFC | 87 | 29 | UUC->UUU | Phe->Phe | CCCGTACTATTTCAATGGTT | C | GTCAGTAGAGATGTTCCCAT | 0.974 |
| ccmFC | 103 | 35 | CCC->UCC | Pro->Ser | GGTTCGTCAGTAGAGATGTT | C | CCATTGGTGCCCCTTCTTCC | 0.992 |
| ccmFC | 119 | 40 | UCU->UUU | Ser->Phe | TGTTCCCATTGGTGCCCCTT | C | TTCCAATGGTACTATAATTC | 0.985 |
| ccmFC | 122 | 41 | UCC->UUC | Ser->Phe | TCCCATTGGTGCCCCTTCTT | C | CAATGGTACTATAATTCCTA | 0.999 |
| ccmFC | 146 | 49 | CCU->CUU | Pro->Leu | TGGTACTATAATTCCTATTC | C | TATCCCTGAATTCCCTTTTT | 0.998 |
| ccmFC | 306 | 102 | UUC->UUU | Phe->Phe | AGAAATGCTTTATTTCGTTT | C | GTTCCCCTTCTTCATTTCCT | 0.91 |
| ccmFC | 312 | 104 | CCC->CCU | Pro->Pro | GCTTTATTTCGTTTCGTTCC | C | CTTCTTCATTTCCTTCTTAT | 0.939 |
| ccmFC | 397 | 133 | CGU->UGU | Arg->Cys | TGCTCTGTTTACAATTCTTT | C | GTACTCTCTTCTCTTTACCA | 0.997 |
| ccmFC | 869 | 290 | UCU->UUU | Ser->Phe | TCATTTACATGGACCCACTT | C | TCATTCCATTTGTGGGAATT | 0.997 |
| ccmFC | 1150 | 384 | CCG->UUG | Pro->Leu | TATTTACGGATCTATATGCT | C | CGATTGGAACTGGAAGTTCC | 0.999 |
| ccmFC | 1151 | 384 | CCG->UUG | Pro->Leu | ATTTACGGATCTATATGCTC | C | GATTGGAACTGGAAGTTCCA | 0.998 |
| ccmFC | 1225 | 409 | CGG->UGG | Arg->Trp | TGCCTTTTATTTTTTGTATT | C | GGATAGGATTTCTGTTGGCT | 0.982 |
| ccmFC | 1250 | 417 | UCG->UUG | Ser->Leu | AGGATTTCTGTTGGCTTCAT | C | GGGAGGCTCGCGTAGTTTGT | 0.986 |
| ccmFC | 1259 | 420 | UCG->UUG | Ser->Leu | GTTGGCTTCATCGGGAGGCT | C | GCGTAGTTTGTTACGTCAGC | 0.981 |
| ccmFC | 1306 | 436 | CGA->UGA | Arg->End | AGGATAAGTTGCATTGGAAT | C | GAGAAAGTTCAGTGGAGTTC | 1 |
| ccmFN | 161 | 54 | CCU->CUU | Pro->Leu | CATTGCTTTGTTTTTCTCTC | C | TTTCCTATCAGCGAGTTCCG | 0.999 |
| ccmFN | 170 | 57 | UCA->UUA | Ser->Leu | GTTTTTCTCTCCTTTCCTAT | C | AGCGAGTTCCGATCCTTTTG | 0.991 |
| ccmFN | 208 | 70 | CGU->UGU | Arg->Cys | TTGTTCGAAATTTCTTCGTT | C | GTACCGAACCGCTTGCAGAA | 0.998 |
| ccmFN | 242 | 81 | CCA->CUA | Pro->Leu | TGCAGAATTAAATCCTGTTC | C | ACAAGATCCTATATCAGCTA | 1 |
| ccmFN | 257 | 86 | UCA->UUA | Ser->Leu | TGTTCCACAAGATCCTATAT | C | AGCTATACATCCTCCTCGCA | 0.967 |
| ccmFN | 274 | 92 | CGC->UGC | Arg->Cys | TATCAGCTATACATCCTCCT | C | GCATTTATGCCGGAGACGTC | 0.999 |
| ccmFN | 406 | 136 | CGC->UGC | Arg->Cys | AAAAGAATGGAACGCTGCTT | C | GCTCTGCTGGATGCGTCGGA | 0.993 |
| ccmFN | 706 | 236 | CGG->UGG | Arg->Trp | GAATTTGGATCTTGACATGT | C | GGTTGTTTTTAACCGTAGGC | 0.994 |
| ccmFN | 734 | 245 | CCA->CUA | Pro->Leu | TTTAACCGTAGGCATCTTGC | C | AGGAAGTTGGTGGGCTCATC | 1 |
| ccmFN | 751 | 251 | CAU->UAU | His->Tyr | TGCCAGGAAGTTGGTGGGCT | C | ATCATGAATTAGGTCGGGGT | 1 |
| ccmFN | 766 | 256 | CGG->UGG | Arg->Trp | GGGCTCATCATGAATTAGGT | C | GGGGTGGCTGGTGGTTTCGG | 0.999 |
| ccmFN | 784 | 262 | CGG->UGG | Arg->Trp | GTCGGGGTGGCTGGTGGTTT | C | GGGATCCTGTAGAAAATGCT | 0.988 |
| ccmFN | 817 | 273 | CGG->UGG | Arg->Trp | AAAATGCTTCTTTTATGCCT | C | GGGTATTAGCTACAGCTCGT | 0.998 |
| ccmFN | 835 | 279 | CGU->UGU | Arg->Cys | CTCGGGTATTAGCTACAGCT | C | GTATTCATTCAGTAATTCTA | 0.998 |
| ccmFN | 878 | 293 | UCG->UUG | Ser->Leu | CCTTCTTCATTCTTGGACCT | C | GCTTCTGAATATTTTTACTC | 0.998 |
| ccmFN | 880 | 294 | CUU->UUU | Leu->Phe | TTCTTCATTCTTGGACCTCG | C | TTCTGAATATTTTTACTCTT | 0.967 |
| ccmFN | 898 | 300 | CUU->UUU | Leu->Phe | CGCTTCTGAATATTTTTACT | C | TTCCATGCTGTGTCTCAGGA | 0.989 |
| ccmFN | 902 | 301 | CCA->CUA | Pro->Leu | TCTGAATATTTTTACTCTTC | C | ATGCTGTGTCTCAGGAACCT | 0.96 |
| ccmFN | 914 | 305 | UCA->UUA | Ser->Leu | TACTCTTCCATGCTGTGTCT | C | AGGAACCTTTTCAATACGGT | 1 |
| ccmFN | 949 | 317 | CCC->UCC | Pro->Ser | TACGGTCCGGATTGCTAGCT | C | CCGTTCATAGTTCTGCTACA | 0.999 |
| ccmFN | 962 | 321 | UCU->UUU | Ser->Phe | GCTAGCTCCCGTTCATAGTT | C | TGCTACAGACGATACACGAG | 1 |
| ccmFN | 997 | 333 | CGG->UGG | Arg->Trp | CACGAGGAAGATTTTTATGG | C | GGTTCTTCCTTCTAATTACA | 0.962 |
| cob | 118 | 40 | CCG->UCG | Pro->Ser | GTTATTGGTGGGGGTTCGGT | C | CGTTAGCTGGTATTAGTTTA | 0.999 |
| cob | 178 | 60 | CAC->UAC | His->Tyr | GCGTTTTTTTAGCTATGCAT | C | ACACACCTCATGTGGATCTA | 0.999 |
| cob | 286 | 96 | CUC->UUC | Leu->Phe | CTAATGGGGCAAGTATGTTT | C | TCATTGTGGTTCACCTTCAT | 0.999 |
| cob | 298 | 100 | CAC->UAC | His->Tyr | GTATGTTTCTCATTGTGGTT | C | ACCTTCATATTTTTCGTGGT | 0.999 |
| cob | 325 | 109 | CAU->UAU | His->Tyr | ATATTTTTCGTGGTCTATAT | C | ATGCGAGTTATAGCAGTCCT | 1 |
| cob | 358 | 120 | CGG->UGG | Arg->Trp | GCAGTCCTAGGGAATTTGTT | C | GGTGTCTCGGAGTTGTCATA | 1 |
| cob | 419 | 140 | CCA->CUA | Pro->Leu | AGCTTTTATAGGATACGTAC | C | ACCTTGGGGTCAGATGAGCT | 1 |
| cob | 564 | 188 | CUC->CUU | Leu->Leu | TTAAATCGTTTTTTTAGTCT | C | CATCATTTACTCCCCTTTAT | 1 |
| cob | 568 | 190 | CAU->UAU | His->Tyr | ATCGTTTTTTTAGTCTCCAT | C | ATTTACTCCCCTTTATTTTA | 0.995 |
| cob | 680 | 227 | UCU->UUU | Ser->Phe | AGAGATGGATAAAATTGCTT | C | TTACCCTTATTTTTATGTAA | 1 |
| cob | 715 | 239 | CGG->UGG | Arg->Trp | ATGTAAAGGATCTAGTAGGT | C | GGGTAGCTTCTGCTATCTTT | 0.998 |
| cob | 725 | 242 | UCU->UUU | Ser->Phe | TCTAGTAGGTCGGGTAGCTT | C | TGCTATCTTTTTTTCCATTT | 0.999 |
| cob | 808 | 270 | CCC->UCC | Pro->Ser | ATATACCCGCTAATCCGATG | C | CCACCCCGCCTCATATTGTG | 1 |
| cob | 853 | 285 | CAC->UAC | His->Tyr | AATGGTATTTCCTACCGATC | C | ACGCCATTCTTCGCAGTATA | 0.997 |
| cob | 908 | 303 | CCA->CUA | Pro->Leu | AGGTGTAGCCGCAATAGCAC | C | AGTTTCTATATCTCTGTTGG | 0.993 |
| cob | 914 | 305 | UCU->UUU | Ser->Phe | AGCCGCAATAGCACCAGTTT | C | TATATCTCTGTTGGCTTTAC | 0.999 |
| cob | 982 | 328 | CAC->UAC | His->Tyr | GTTCAAGTTTTCGCCCGATT | C | ACCAAGGAATATTTTGGTTG | 0.999 |
| cob | 1015 | 339 | CGC->UGC | Arg->Cys | TTTGGTTGCTTTTGGCGGAT | C | GCTTACTACTAGGTTGGATC | 0.999 |
| cob | 1081 | 361 | CCU->UCU | Pro->Ser | TTGTGACTATTGGACAAATT | C | CTTCCGTCTTTTTCTTCTTG | 0.989 |
| cob | 1124 | 375 | CCG->CUG | Pro->Leu | CTTTGCCATAACGCCCATTC | C | GGGATGGGTTGGAAGAGGAA | 0.996 |
| cox1 | 188 | 63 | UCU->UUU | Ser->Phe | GTTAATAACGGCTCACGCTT | C | TTTAATGATCTTTTTTATGG | 0.998 |
| cox1 | 233 | 78 | UCU->UUU | Ser->Phe | GCCGGCGATGATAGGTGGAT | C | TGGTAATTGGTTTGTTCCTC | 0.998 |
| cox1 | 443 | 148 | UCU->UUU | Ser->Phe | AGCAGTTGATTTAGCAATTT | C | TAGTCTTCATCTATCCGGTG | 1 |
| cox1 | 542 | 181 | UCA->UUA | Ser->Leu | TGGAATGACTATGCATAGAT | C | ACCCCTTTTTGTCTGGTCCG | 0.996 |
| cox1 | 659 | 220 | UCU->UUU | Ser->Phe | AAACTTTAATACAACCTTTT | C | TGATCCAGCTGGCGGGGGCG | 0.991 |
| cox1 | 737 | 246 | CCC->CUC | Pro->Leu | TCATCCAGAGGTGTATATTC | C | CATTCTGCCTGGATTCGGTA | 0.999 |
| cox1 | 1393 | 465 | CGU->UGU | Arg->Cys | ATATATCCGTAGTTGGGATT | C | GTCGTTTCTTCGTGGTCGTA | 0.997 |
| cox1 | 1396 | 466 | CGU->UGU | Arg->Cys | TATCCGTAGTTGGGATTCGT | C | GTTTCTTCGTGGTCGTAACA | 1 |
| cox1 | 1424 | 475 | UCA->UUA | Ser->Leu | CGTGGTCGTAACAATCACTT | C | AAGCAGTGGAAACAACAAAA | 0.997 |
| cox1 | 1437 | 479 | AAC->AAU | Asn->Asn | ATCACTTCAAGCAGTGGAAA | C | AACAAAAGATGTGCTCCAAG | 0.997 |
| cox1 | 1480 | 494 | CCA->UCA | Pro->Ser | CTTGGGCTGTTGAACAGAAT | C | CAACCACACCTGAATGGATG | 0.996 |
| cox1 | 1490 | 497 | CCU->CUU | Pro->Leu | TGAACAGAATCCAACCACAC | C | TGAATGGATGGTACAAAGTC | 0.999 |
| cox2 | 27 | 9 | CUC->CUU | Leu->Leu | GTTCTAGAATGGCTCTTCCT | C | ACAATTGCTCCTTGTGATGC | 0.953 |
| cox2 | 71 | 24 | UCU->UUU | Ser->Phe | GGAACCATGGCAATTAGGAT | C | TCAAGACGGAGCAACACCTA | 0.997 |
| cox2 | 161 | 54 | UCA->UUA | Ser->Leu | TCTGATTTTGGTTTTCGTAT | C | ACGGATCTTGGTTCGCGCTT | 0.993 |
| cox2 | 163 | 55 | CGG->UGG | Arg->Trp | TGATTTTGGTTTTCGTATCA | C | GGATCTTGGTTCGCGCTTTA | 1 |
| cox2 | 443 | 148 | ACG->AUG | Thr->Met | ACTCACTTTTGACAGTTATA | C | GATTCCAGAAGATGATCCAG | 0.999 |
| cox2 | 460 | 154 | CCA->UUA | Pro->Leu | ATACGATTCCAGAAGATGAT | C | CAGAATTGGGTCAATTACGT | 0.917 |
| cox2 | 461 | 154 | CCA->UUA | Pro->Leu | TACGATTCCAGAAGATGATC | C | AGAATTGGGTCAATTACGTT | 0.999 |
| cox2 | 544 | 182 | CCU->UCU | Pro->Ser | ATCTACGTATGATTGTAACA | C | CTGCTGATGTACCTCATAGT | 0.999 |
| cox2 | 557 | 186 | CCU->CUU | Pro->Leu | TGTAACACCTGCTGATGTAC | C | TCATAGTTGGGCTGTACCTT | 0.996 |
| cox2 | 581 | 194 | UCA->UUA | Ser->Leu | TAGTTGGGCTGTACCTTCCT | C | AGGTGTCAAATGTGATGCCG | 1 |
| cox2 | 614 | 205 | UCA->UUA | Ser->Leu | TGATGCCGTACCTGGTCGTT | C | AAATCAGACCTCCATTTCGG | 0.993 |
| cox2 | 632 | 211 | UCG->UUG | Ser->Leu | TTCAAATCAGACCTCCATTT | C | GGTACAACGAGAAGGAGTTT | 0.99 |
| cox2 | 676 | 226 | CGU->UGU | Arg->Cys | ATGGTCAGTGCAGTGAGATT | C | GTGGAACTAATCATGCCTTT | 0.999 |
| cox2 | 698 | 233 | ACG->AUG | Thr->Met | TGGAACTAATCATGCCTTTA | C | GCCTATCGTCGTAGAAGCAG | 0.994 |
| cox2 | 721 | 241 | CCU->UCU | Pro->Ser | CTATCGTCGTAGAAGCAGTT | C | CTTTGAAAGATTATGGTTCT | 0.999 |
| cox2 | 742 | 248 | CGG->UGG | Arg->Trp | CTTTGAAAGATTATGGTTCT | C | GGGTATCCAATCAATTAATC | 0.997 |
| cox3 | 112 | 38 | CCA->UCA | Pro->Ser | GAGGTGTGATGTACATGCAC | C | CATTTCAAGGGGGTGCAACA | 1 |
| cox3 | 245 | 82 | CCU->CUU | Pro->Leu | AAAAGCTGTACAATTAGGAC | C | TCGATATGGTTCTATTCTGT | 1 |
| cox3 | 257 | 86 | UCU->UUU | Ser->Phe | ATTAGGACCTCGATATGGTT | C | TATTCTGTTCATAGTCTCGG | 0.994 |
| cox3 | 289 | 97 | CUU->UUU | Leu->Phe | TAGTCTCGGAGGTTATGTTC | C | TTTTTGCTTTTTTTTGGGCT | 0.999 |
| cox3 | 311 | 104 | UCU->UUU | Ser->Phe | TTTTGCTTTTTTTTGGGCTT | C | TTCTCATTCTTCTTTGGCAC | 0.999 |
| cox3 | 314 | 105 | UCU->UUU | Ser->Phe | TGCTTTTTTTTGGGCTTCTT | C | TCATTCTTCTTTGGCACCTA | 0.999 |
| cox3 | 388 | 130 | CGG->UGG | Arg->Trp | GAATTGGGGTTTTAGATCCT | C | GGGAAATTCCTTTTCTTAAT | 0.996 |
| cox3 | 413 | 138 | CCU->CUU | Pro->Leu | AATTCCTTTTCTTAATACCC | C | TATTCTCCCTTCATCCGGAG | 0.994 |
| cox3 | 422 | 141 | CCU->CUU | Pro->Leu | TCTTAATACCCCTATTCTCC | C | TTCATCCGGAGCTGCCGTAA | 0.993 |
| cox3 | 512 | 171 | UCA->UUA | Ser->Leu | CGCTTTAGTAGCTACCGTTT | C | ACTGGCTCTAGTATCCACTG | 0.999 |
| cox3 | 527 | 176 | UCC->UUC | Ser->Phe | CGTTTCACTGGCTCTAGTAT | C | CACTGTCTTTCAAGGAATGG | 1 |
| cox3 | 566 | 189 | UCC->UUC | Ser->Phe | GGAATATTATCAAGCACCCT | C | CACTCTTTCGGATAGTATTT | 0.998 |
| cox3 | 605 | 202 | UCA->UUA | Ser->Leu | TTATGGTTCTACCTTTTTCT | C | AGCAACTGGCTTTCATGGTT | 1 |
| cox3 | 754 | 252 | CGG->UGG | Arg->Trp | GGCATTTTGTAGACGTGGTT | C | GGTTATTCCCATTTGTCTCT | 1 |
| cox3 | 764 | 255 | CCA->CUA | Pro->Leu | AGACGTGGTTCGGTTATTCC | C | ATTTGTCTCTATCTATTGGT | 1 |
| matR | 8 | 3 | CCC->CUC | Pro->Leu | AGAGGCGATCAGAATGGTAC | C | CGAATCCATTTACGATCCCG | 1 |
| matR | 14 | 5 | UCC->UUC | Ser->Phe | GATCAGAATGGTACCCGAAT | C | CATTTACGATCCCGAGTTTA | 0.926 |
| matR | 157 | 53 | CCA->UCA | Pro->Ser | TCGACCGACATCGATTCATC | C | CAATCTTTAAGGAAGAGATC | 0.984 |
| matR | 290 | 97 | CCA->CUA | Pro->Leu | TGTACTACTATCGGCCCTAC | C | AGGCAACATCTACCTACACA | 0.995 |
| matR | 346 | 116 | CAC->UAC | His->Tyr | TAGGGAGGATCCGACAGAAG | C | ACGAAATTCCTCTTATTCTT | 0.995 |
| matR | 986 | 329 | CCC->CUC | Pro->Leu | ACGGAGTACGGTAGAATTCC | C | CGGTACGGTCATTCGGGAAG | 0.999 |
| matR | 1396 | 466 | CGG->UGG | Arg->Trp | TCCGGAAGTTTTCATTAGGT | C | GGAAGGCGGCGGGCGAAGGT | 0.955 |
| matR | 1754 | 585 | UCC->UUC | Ser->Phe | CGGAGACATAGTAAATTGGT | C | CGCGGGCATCGCAATAAGTC | 0.985 |
| matR | 1775 | 592 | CCU->CUU | Pro->Leu | CGCGGGCATCGCAATAAGTC | C | TCTGTCCTACTACAGGTGCC | 0.998 |
| matR | 1795 | 599 | CGC->UGC | Arg->Cys | CTCTGTCCTACTACAGGTGC | C | GCGACAACCTTTACCAAGTC | 1 |
| matR | 1809 | 603 | UAC->UAU | Tyr->Tyr | AGGTGCCGCGACAACCTTTA | C | CAAGTCCGAACGATTGTCGA | 0.996 |
| matR | 1831 | 611 | CAC->UAC | His->Tyr | AAGTCCGAACGATTGTCGAC | C | ACCAGATCCGCTGGTCCACT | 0.969 |
| matR | 1901 | 634 | CCA->CUA | Pro->Leu | TTCGGCGCGGAATATAATCC | C | AAAGTACCCCAAAGACTCAA | 0.996 |
| matR | 1909 | 637 | CCC->UCC | Pro->Ser | GGAATATAATCCCAAAGTAC | C | CCAAAGACTCAAATATAGTA | 1 |
| matR | 1919 | 640 | UCA->UUA | Ser->Leu | CCCAAAGTACCCCAAAGACT | C | AAATATAGTAAGTAAAGAAG | 0.998 |
| mttB | 20 | 7 | CCG->CUG | Pro->Leu | CATTGAATTAAATTTCGCAC | C | GGAAACGATTTTAGGAGAAG | 0.922 |
| mttB | 53 | 18 | UCU->UUU | Ser->Phe | AGGAGAAGTTCGAATCCGTT | C | TCTTCGGATATTGATCGGTC | 0.941 |
| mttB | 58 | 20 | CGG->UGG | Arg->Trp | AAGTTCGAATCCGTTCTCTT | C | GGATATTGATCGGTCTTGGT | 0.976 |
| mttB | 94 | 32 | CGU->UGU | Arg->Cys | TTGGTTTGACATGGTTTACG | C | GTTACAGGTTCTCGGAAGAG | 0.982 |
| mttB | 125 | 42 | CCA->CUA | Pro->Leu | CTCGGAAGAGTTAATTTTTC | C | ATTAGCTAAACCCTTTCTGA | 0.993 |
| mttB | 172 | 58 | CGU->UGU | Arg->Cys | CTTTGGACTCGTATTTTGTT | C | GTACACAATCAACGGAGGCC | 0.998 |
| mttB | 182 | 61 | UCA->UUA | Ser->Leu | GTATTTTGTTCGTACACAAT | C | AACGGAGGCCTCCCCGACAT | 1 |
| mttB | 194 | 65 | UCC->UUC | Ser->Phe | TACACAATCAACGGAGGCCT | C | CCCGACATATGTTGCAACGT | 1 |
| mttB | 196 | 66 | CCG->UCG | Pro->Ser | CACAATCAACGGAGGCCTCC | C | CGACATATGTTGCAACGTCT | 0.999 |
| mttB | 217 | 73 | CCA->UCA | Pro->Ser | CGACATATGTTGCAACGTCT | C | CAATAGCATGCTCTTACTTC | 1 |
| mttB | 256 | 86 | CAU->UAU | His->Tyr | TCGTCTTTCCCTTAATAAGT | C | ATCAAATTTGGTGCTTTTCG | 0.989 |
| mttB | 275 | 92 | UCG->UUG | Ser->Leu | TCATCAAATTTGGTGCTTTT | C | GATCCCCAGTTGCTATGGGG | 0.997 |
| mttB | 322 | 108 | CUC->UUC | Leu->Phe | GGACGAAATACAATCGATTC | C | TCCATTTAAGTGGTTCTCGC | 0.984 |
| mttB | 325 | 109 | CAU->UAU | His->Tyr | CGAAATACAATCGATTCCTC | C | ATTTAAGTGGTTCTCGCTTC | 0.983 |
| mttB | 338 | 113 | UCU->UUU | Ser->Phe | ATTCCTCCATTTAAGTGGTT | C | TCGCTTCTCCCTGTTCTTGT | 0.978 |
| mttB | 340 | 114 | CGC->UGC | Arg->Cys | TCCTCCATTTAAGTGGTTCT | C | GCTTCTCCCTGTTCTTGTTC | 0.999 |
| mttB | 367 | 123 | CCU->UUU | Pro->Phe | CCCTGTTCTTGTTCCTAACT | C | CTCCCCGGGTAGTTCCCAAT | 0.955 |
| mttB | 368 | 123 | CCU->UUU | Pro->Phe | CCTGTTCTTGTTCCTAACTC | C | TCCCCGGGTAGTTCCCAATG | 0.998 |
| mttB | 370 | 124 | CCC->UCC | Pro->Ser | TGTTCTTGTTCCTAACTCCT | C | CCCGGGTAGTTCCCAATGTT | 0.989 |
| mttB | 373 | 125 | CGG->UGG | Arg->Trp | TCTTGTTCCTAACTCCTCCC | C | GGGTAGTTCCCAATGTTTGG | 0.997 |
| mttB | 401 | 134 | CCA->CUA | Pro->Leu | TCCCAATGTTTGGCACTTTC | C | ATACTTCGTGGGTGCAACAT | 0.999 |
| mttB | 491 | 164 | UCG->UUG | Ser->Leu | TATGTTAACTGTTCGTATTT | C | GTTCATTCCATCGGTATGCT | 0.994 |
| mttB | 499 | 167 | CCA->UCA | Pro->Ser | CTGTTCGTATTTCGTTCATT | C | CATCGGTATGCTCTCAGGTA | 0.978 |
| mttB | 535 | 179 | CGU->UGU | Arg->Cys | AGGTACCTGTAATTGTGATC | C | GTTTGCCAGAACCAAGGGGT | 0.997 |
| mttB | 542 | 181 | CCA->CUA | Pro->Leu | TGTAATTGTGATCCGTTTGC | C | AGAACCAAGGGGTCTTTCTG | 0.999 |
| mttB | 548 | 183 | CCA->CUA | Pro->Leu | TGTGATCCGTTTGCCAGAAC | C | AAGGGGTCTTTCTGTGGAAA | 0.998 |
| mttB | 604 | 202 | CCG->UCG | Pro->Ser | GCCGTTTTTTGATGGTTTTT | C | CGCTTATCACAGCTGCTCTT | 0.988 |
| mttB | 661 | 221 | CCU->UCU | Pro->Ser | TCTGGTGCCAAATCGTCGCC | C | CTTTCCTTATTTCTTTGATA | 0.98 |
| nad1 | 215 | 72 | UCC->UUC | Ser->Phe | ACCAAGTAGTGCTAATTTCT | C | CCTTTTTCGAATGGCTCCAG | 0.999 |
| nad1 | 265 | 89 | CGG->UGG | Arg->Trp | TTATGTTAAGTCTGGTCGCT | C | GGGCCGTTGTACCTTTTGAT | 0.999 |
| nad1 | 307 | 103 | CCG->UUG | Pro->Leu | ATGGTATGGTATTGTCAGAT | C | CGAACATAGGGCTACTTTAT | 0.995 |
| nad1 | 308 | 103 | CCG->UUG | Pro->Leu | TGGTATGGTATTGTCAGATC | C | GAACATAGGGCTACTTTATT | 1 |
| nad1 | 436 | 146 | CCU->UCU | Pro->Ser | GATCTGCAGCTCAAATGGTC | C | CTTATGAAGTCTCTATTGGT | 0.998 |
| nad1 | 490 | 164 | CCC->UCU | Pro->Ser | CTGTACTAATATGTGTAGGT | C | CCTGTAATTCGAGTGAGATT | 1 |
| nad1 | 492 | 164 | CCC->UCU | Pro->Ser | GTACTAATATGTGTAGGTCC | C | TGTAATTCGAGTGAGATTGT | 0.987 |
| nad1 | 500 | 167 | UCG->UUG | Ser->Leu | ATGTGTAGGTCCCTGTAATT | C | GAGTGAGATTGTCATGGCGC | 1 |
| nad1 | 536 | 179 | UCC->UUU | Ser->Phe | GGCGCAAAAGCAGATATGGT | C | CGGTATTCCCTTGTTCCCCG | 0.997 |
| nad1 | 537 | 179 | UCC->UUU | Ser->Phe | GCGCAAAAGCAGATATGGTC | C | GGTATTCCCTTGTTCCCCGT | 0.988 |
| nad1 | 555 | 185 | CCC->CCU | Pro->Pro | TCCGGTATTCCCTTGTTCCC | C | GTATTGGTTATGTTCTTTAT | 0.996 |
| nad1 | 577 | 193 | CCU->UCU | Pro->Ser | TATTGGTTATGTTCTTTATT | C | CTCGTCTAGCAGAAACTAAT | 1 |
| nad1 | 580 | 194 | CGU->UGU | Arg->Cys | TGGTTATGTTCTTTATTCCT | C | GTCTAGCAGAAACTAATCGA | 1 |
| nad1 | 608 | 203 | UCU->UUU | Ser->Phe | AGAAACTAATCGAGCTCCGT | C | TGATCTCCCAGAAGCGGAAG | 1 |
| nad1 | 635 | 212 | UCA->UUA | Ser->Leu | CCCAGAAGCGGAAGCTGAAT | C | AGTTGCAGGCTATAATGTAG | 0.999 |
| nad1 | 674 | 225 | UCU->UUU | Ser->Phe | AGAATATTCTTCAATGGGGT | C | TGCTCTTTTTTTTTTGGGAG | 1 |
| nad1 | 725 | 242 | CCA->CUA | Pro->Leu | TATGATCTTAATGAGCGGTC | C | ATGCACATTGCTCTTTCCAG | 0.993 |
| nad1 | 743 | 248 | CCA->CUA | Pro->Leu | TCCATGCACATTGCTCTTTC | C | AGGAGGTTGGCCGCCTATCC | 0.999 |
| nad1 | 755 | 252 | CCG->CUG | Pro->Leu | GCTCTTTCCAGGAGGTTGGC | C | GCCTATCCTAGATCTTCCCA | 0.996 |
| nad1 | 779 | 260 | UCC->UUC | Ser->Phe | TATCCTAGATCTTCCCATTT | C | CAAGAAGATCCCGGGCTCGA | 1 |
| nad1 | 823 | 275 | CUU->UUU | Leu->Phe | GGTTTAGTATCAAGGTAATT | C | TTTTTCTGTTCCTATATATA | 0.918 |
| nad1 | 834 | 278 | UUC->UUU | Phe->Phe | AAGGTAATTCTTTTTCTGTT | C | CTATATATATGGGTCCGTGC | 0.968 |
| nad1 | 893 | 298 | CCU->CUU | Pro->Leu | TTATGATCAATTAATGGGAC | C | TGGCCGGAAAGTGTTCTTGC | 1 |
| nad1 | 898 | 300 | CGG->UGG | Arg->Trp | ATCAATTAATGGGACCTGGC | C | GGAAAGTGTTCTTGCCCCTA | 0.999 |
| nad1 | 928 | 310 | CGG->UGG | Arg->Trp | TCTTGCCCCTATCATTAGCT | C | GGGTAGTCCCCGTTTCTGGT | 0.998 |
| nad1 | 937 | 313 | CCC->UCC | Pro->Ser | TATCATTAGCTCGGGTAGTC | C | CCGTTTCTGGTGTTTCAGTC | 0.993 |
| nad1 | 953 | 318 | UCA->UUA | Ser->Leu | AGTCCCCGTTTCTGGTGTTT | C | AGTCACCTTTCGATGGCTCC | 0.997 |
| nad2 | 26 | 9 | UCC->UUC | Ser->Phe | CAATCTTTTTGTAGCGGTTT | C | CCCAGAGATCTTTATCATTA | 0.987 |
| nad2 | 800 | 267 | UCU->UUU | Ser->Phe | TGCGCCTAAAATCTCTATTT | C | TGCAAATATGTCACGTCTTT | 0.968 |
| nad2 | 812 | 271 | UCA->UUA | Ser->Leu | CTCTATTTCTGCAAATATGT | C | ACGTCTTTCTATTGTTGGTT | 0.916 |
| nad2 | 821 | 274 | UCU->UUU | Ser->Phe | TGCAAATATGTCACGTCTTT | C | TATTGTTGGTTCCTATGGAG | 0.99 |
| nad3 | 44 | 15 | CCG->CUG | Pro->Leu | TATCTATTTAGCGATCAGTC | C | GCTAGTTTCTTTGATCCCAC | 1 |
| nad3 | 62 | 21 | CCA->CUA | Pro->Leu | TCCGCTAGTTTCTTTGATCC | C | ACTCGGTCTTCCTTTTCCAT | 0.999 |
| nad3 | 80 | 27 | CCA->CUA | Pro->Leu | CCCACTCGGTCTTCCTTTTC | C | ATTTGCTTCCAATAGTTCGA | 0.998 |
| nad3 | 124 | 42 | CAC->UAC | His->Tyr | ATCCAGAAAAATTGTCGGCC | C | ACGAATGTGGTTTCGATCCC | 0.999 |
| nad3 | 146 | 49 | UCC->UUC | Ser->Phe | CGAATGTGGTTTCGATCCCT | C | CGGTGATGCCAGAAGTCGTT | 0.999 |
| nad3 | 185 | 62 | CCG->CUG | Pro->Leu | TTTCGATATACGATTTTATC | C | GGTTCCTATTTTATTTATTA | 0.998 |
| nad3 | 190 | 64 | CCU->UCU | Pro->Ser | ATATACGATTTTATCCGGTT | C | CTATTTTATTTATTATCCCT | 1 |
| nad3 | 208 | 70 | CCU->UUU | Pro->Phe | TTCCTATTTTATTTATTATC | C | CTGATCCGGAAGTCACCTTT | 0.997 |
| nad3 | 209 | 70 | CCU->UUU | Pro->Phe | TCCTATTTTATTTATTATCC | C | TGATCCGGAAGTCACCTTTT | 0.999 |
| nad3 | 215 | 72 | CCG->CUG | Pro->Leu | TTTATTTATTATCCCTGATC | C | GGAAGTCACCTTTTCTTCTC | 0.995 |
| nad3 | 230 | 77 | UCU->UUU | Ser->Phe | TGATCCGGAAGTCACCTTTT | C | TTCTCCTTGGGCAGTACCTC | 0.997 |
| nad3 | 233 | 78 | UCU->UUU | Ser->Phe | TCCGGAAGTCACCTTTTCTT | C | TCCTTGGGCAGTACCTCCCA | 0.997 |
| nad3 | 247 | 83 | CCU->UCU | Pro->Ser | TTTCTTCTCCTTGGGCAGTA | C | CTCCCAACAAGATTGATCTG | 0.993 |
| nad3 | 251 | 84 | CCC->CUC | Pro->Leu | TTCTCCTTGGGCAGTACCTC | C | CAACAAGATTGATCTGTTTG | 0.998 |
| nad3 | 275 | 92 | UCU->UUU | Ser->Phe | CAAGATTGATCTGTTTGGAT | C | TTGGTCCATGATGGCCTTTT | 0.995 |
| nad3 | 317 | 106 | UCU->UUU | Ser->Phe | ATTGATTTTGACGATTGGAT | C | TCTCTATGAATGGAAAAGGG | 1 |
| nad3 | 344 | 115 | UCG->UUG | Ser->Leu | TGAATGGAAAAGGGGTGCTT | C | GGATCGGGAGTAACCACTAG | 0.999 |
| nad3 | 349 | 117 | CGG->UGG | Arg->Trp | GGAAAAGGGGTGCTTCGGAT | C | GGGAGTAACCACTAGTGATA | 0.999 |
| nad4 | 29 | 10 | UCC->UUC | Ser->Phe | ACATTTCTGTGAATGCTATT | C | CGATCTAAGTGGTCCTATTC | 1 |
| nad4 | 44 | 15 | CCU->CUU | Pro->Leu | CTATTCCGATCTAAGTGGTC | C | TATTCTGTGTCCCGTGCTAG | 0.998 |
| nad4 | 74 | 25 | ACU->AUU | Thr->Ile | TCCCGTGCTAGGAAGCATTA | C | TCCTCTTTTCATTCCAAATT | 0.997 |
| nad4 | 77 | 26 | CCU->CUU | Pro->Leu | CGTGCTAGGAAGCATTACTC | C | TCTTTTCATTCCAAATTCAA | 0.999 |
| nad4 | 107 | 36 | CCG->CUG | Pro->Leu | TCCAAATTCAAGAATACGAC | C | GATACGATTGATTGGTCTGT | 0.999 |
| nad4 | 154 | 52 | CCC->UCC | Pro->Ser | CTCTTATTACTTTTTTGTAT | C | CCCCTGTTCTTCGGATACAA | 1 |
| nad4 | 158 | 53 | CCU->CUU | Pro->Leu | TATTACTTTTTTGTATCCCC | C | TGTTCTTCGGATACAATTCG | 0.997 |
| nad4 | 166 | 56 | CGG->UGG | Arg->Trp | TTTTGTATCCCCCTGTTCTT | C | GGATACAATTCGATCCTTCT | 0.992 |
| nad4 | 197 | 66 | UCU->UUU | Ser->Phe | CGATCCTTCTACGGCCAAAT | C | TCAATTTGTGGAAAGCCTTC | 0.998 |
| nad4 | 317 | 106 | UCA->UUA | Ser->Leu | TCTGATCCCTATTTGCATTT | C | AGTGGGTTGGTCTAGTATGA | 0.999 |
| nad4 | 362 | 121 | ACA->AUA | Thr->Ile | TTATGGGAAAGAGTATATAA | C | AGCATCTCTAATTCGTGAAT | 0.997 |
| nad4 | 368 | 123 | UCU->UUU | Ser->Phe | GAAAGAGTATATAACAGCAT | C | TCTAATTCGTGAATTTCTAA | 0.999 |
| nad4 | 376 | 126 | CGU->UGU | Arg->Cys | ATATAACAGCATCTCTAATT | C | GTGAATTTCTAATGATCGCC | 0.967 |
| nad4 | 401 | 134 | UCC->UUC | Ser->Phe | ATTTCTAATGATCGCCGTGT | C | CTGCATGCTGGATCCTCTAC | 1 |
| nad4 | 416 | 139 | CCU->CUU | Pro->Leu | CGTGTCCTGCATGCTGGATC | C | TCTACTATTCTATGTTCTTC | 0.998 |
| nad4 | 433 | 145 | CUU->UUU | Leu->Phe | ATCCTCTACTATTCTATGTT | C | TTCCCGAAAGCGTGCCAATC | 0.999 |
| nad4 | 436 | 146 | CCC->UUC | Pro->Phe | CTCTACTATTCTATGTTCTT | C | CCGAAAGCGTGCCAATCCCT | 0.999 |
| nad4 | 437 | 146 | CCC->UUC | Pro->Phe | TCTACTATTCTATGTTCTTC | C | CGAAAGCGTGCCAATCCCTA | 0.992 |
| nad4 | 449 | 150 | CCA->CUA | Pro->Leu | TGTTCTTCCCGAAAGCGTGC | C | AATCCCTATGTTCATTATTA | 0.995 |
| nad4 | 533 | 178 | UCA->UUA | Ser->Leu | TCAGTTTTTCCTATATACTT | C | ACTTGGATCCGTTTTTATGC | 0.998 |
| nad4 | 566 | 189 | CCG->CUG | Pro->Leu | TTTTATGCTATTAGCTATTC | C | GTTGATTCTTCTCCAAACAG | 0.999 |
| nad4 | 767 | 256 | CCU->CUU | Pro->Leu | CGTCATCTTGGCAGGAATTC | C | TTTAAAATTGGGAACCTACG | 0.999 |
| nad4 | 832 | 278 | CGU->UGU | Arg->Cys | TGTTTCCCGAAGCGACACTT | C | GTTCCACTCCTTTCATTTAT | 0.999 |
| nad4 | 836 | 279 | UCC->UUC | Ser->Phe | TCCCGAAGCGACACTTCGTT | C | CACTCCTTTCATTTATACTC | 0.992 |
| nad4 | 856 | 286 | CCA->UUA | Pro->Leu | CCACTCCTTTCATTTATACT | C | CAAGCGCGATTGCTATAATA | 0.979 |
| nad4 | 857 | 286 | CCA->UUA | Pro->Leu | CACTCCTTTCATTTATACTC | C | AAGCGCGATTGCTATAATAT | 1 |
| nad4 | 883 | 295 | CCC->UCC | Pro->Ser | CGATTGCTATAATATATACT | C | CCTCGACCACTTCAAGACAG | 1 |
| nad4 | 887 | 296 | UCG->UUG | Ser->Leu | TGCTATAATATATACTCCCT | C | GACCACTTCAAGACAGATTG | 0.998 |
| nad4 | 896 | 299 | UCA->UUA | Ser->Leu | ATATACTCCCTCGACCACTT | C | AAGACAGATTGATCTTAAGA | 0.995 |
| nad4 | 934 | 312 | CCU->UCU | Pro->Ser | AGAAGATCATTGCCTACTCC | C | CTGTAGCCCATATGAATCTG | 0.999 |
| nad4 | 971 | 324 | UCU->UUU | Ser->Phe | TCTGGTGACTATTGGTATGT | C | TAGTCCGAACATACAGGGAA | 1 |
| nad4 | 977 | 326 | CCG->CUG | Pro->Leu | GACTATTGGTATGTCTAGTC | C | GAACATACAGGGAATTGGAG | 1 |
| nad4 | 1006 | 336 | CCA->UUA | Pro->Leu | AGGGAATTGGAGGTAGCATT | C | CACCGATGTCAAGTCATGGA | 0.986 |
| nad4 | 1007 | 336 | CCA->UUA | Pro->Leu | GGGAATTGGAGGTAGCATTC | C | ACCGATGTCAAGTCATGGAC | 0.992 |
| nad4 | 1010 | 337 | CCG->CUG | Pro->Leu | AATTGGAGGTAGCATTCCAC | C | GATGTCAAGTCATGGACTGG | 1 |
| nad4 | 1016 | 339 | UCA->UUA | Ser->Leu | AGGTAGCATTCCACCGATGT | C | AAGTCATGGACTGGTTCCTT | 1 |
| nad4 | 1033 | 345 | CCU->UCU | Pro->Ser | TGTCAAGTCATGGACTGGTT | C | CTTCAGCCCTTTTTCTATGT | 0.999 |
| nad4 | 1129 | 377 | CUC->UUC | Leu->Phe | TAGTGAGCACCATGCCGAAT | C | TCCCTACCATTTCCTTCTCT | 0.998 |
| nad4 | 1132 | 378 | CCU->UCU | Pro->Ser | TGAGCACCATGCCGAATCTC | C | CTACCATTTCCTTCTCTTCC | 0.998 |
| nad4 | 1142 | 381 | UCC->UUC | Ser->Phe | GCCGAATCTCCCTACCATTT | C | CTTCTCTTCCACTTTGGCCA | 0.99 |
| nad4 | 1148 | 383 | UCU->UUU | Ser->Phe | TCTCCCTACCATTTCCTTCT | C | TTCCACTTTGGCCAATATGA | 0.998 |
| nad4 | 1151 | 384 | UCC->UUC | Ser->Phe | CCCTACCATTTCCTTCTCTT | C | CACTTTGGCCAATATGAGTT | 0.992 |
| nad4 | 1205 | 402 | CCA->CUA | Pro->Leu | CAGCTTTATCGGGGAATTTC | C | AATCTCAGTAGGATCTTTCC | 0.99 |
| nad4 | 1211 | 404 | UCA->UUA | Ser->Leu | TATCGGGGAATTTCCAATCT | C | AGTAGGATCTTTCCAAAGAA | 0.973 |
| nad4 | 1307 | 436 | GCG->GUG | Ala->Val | CCTTTGGCTATATAATCGCG | C | GGTTTCTGGAAATTTAAAAC | 0.994 |
| nad4 | 1355 | 452 | CCA->CUA | Pro->Leu | CCTCCATAAATTCTCCGATC | C | AAATGGCAGAGAAGTTTCCA | 0.995 |
| nad4 | 1373 | 458 | UCC->UUC | Ser->Phe | TCCAAATGGCAGAGAAGTTT | C | CATCTTTCTACCTTTTCTTC | 0.993 |
| nad4L | 2 | 1 | ACG->AUG | Thr->Met | GACATTCCACGTTTCCGAAA | C | GGATCCTATCAAATATTTCA | 1 |
| nad4L | 41 | 14 | UCU->UUU | Ser->Phe | CACATTTTCGATGATCATCT | C | TATTTTAGGTATTCGGGGAA | 0.997 |
| nad4L | 55 | 19 | CGG->UGG | Arg->Trp | TCATCTCTATTTTAGGTATT | C | GGGGAATCCTCCTTAATAGA | 0.998 |
| nad4L | 86 | 29 | CCU->CUU | Pro->Leu | CCTTAATAGACGAAATATTC | C | TATTATGCCAATGCCAATTG | 0.957 |
| nad4L | 95 | 32 | CCA->CUA | Pro->Leu | ACGAAATATTCCTATTATGC | C | AATGCCAATTGAATCAATGT | 0.996 |
| nad4L | 100 | 34 | CCA->UCA | Pro->Ser | ATATTCCTATTATGCCAATG | C | CAATTGAATCAATGTTATTA | 0.999 |
| nad4L | 110 | 37 | UCA->UUA | Ser->Leu | TATGCCAATGCCAATTGAAT | C | AATGTTATTAGCTGTCAATT | 0.998 |
| nad4L | 131 | 44 | UCG->UUG | Ser->Leu | AATGTTATTAGCTGTCAATT | C | GAACTTTTTGGTCTTTTCCG | 1 |
| nad4L | 179 | 60 | UCA->UUA | Ser->Leu | GGATGATATGATGGGTCAAT | C | ATTTGCTTCATTAGTTCCAA | 1 |
| nad4L | 188 | 63 | UCA->UUA | Ser->Leu | GATGGGTCAATCATTTGCTT | C | ATTAGTTCCAACAGTAGCAG | 0.995 |
| nad4L | 197 | 66 | CCA->CUA | Pro->Leu | ATCATTTGCTTCATTAGTTC | C | AACAGTAGCAGCTGCGGAAT | 0.995 |
| nad4L | 230 | 77 | UCA->UUA | Ser->Leu | TGCGGAATCCGCTATTGGAT | C | AGCCATTTTCGTTATTACTT | 0.999 |
| nad4L | 281 | 94 | UCC->UUC | Ser->Phe | AGGGACTATTGCTGTCGAAT | C | CATAAATTGCATTCAAGGTT | 0.998 |
| nad5 | 155 | 52 | CCG->CUG | Pro->Leu | TGCTTTTTATGAAGTAGCAC | C | GGGAGCTAGTGCTTGCTATC | 0.999 |
| nad5 | 242 | 81 | CCG->CUG | Pro->Leu | GGGCTTCTTGTTCGATAGCC | C | GACCGTAGTGATGTTAATTG | 1 |
| nad5 | 272 | 91 | CCC->CUC | Pro->Leu | GATGTTAATTGTGGTTACAC | C | CATAAGTAGCTTGGTCCATC | 0.989 |
| nad5 | 315 | 105 | CCC->CCU | Pro->Pro | TATTCCATTTCATATATGCC | C | GAGGATCCGCATAGCCCTCG | 0.954 |
| nad5 | 358 | 120 | CCU->UUU | Pro->Phe | TTATGTGTTATTTATCCATT | C | CTACTTTTTTTATGCCAATG | 0.994 |
| nad5 | 359 | 120 | CCU->UUU | Pro->Phe | TATGTGTTATTTATCCATTC | C | TACTTTTTTTATGCCAATGT | 0.999 |
| nad5 | 374 | 125 | CCA->CUA | Pro->Leu | CATTCCTACTTTTTTTATGC | C | AATGTTGGTGACTGGAGATA | 0.996 |
| nad5 | 398 | 133 | UCU->UUU | Ser->Phe | GTTGGTGACTGGAGATAACT | C | TCTTCAATTATTCCTGGGAT | 1 |
| nad5 | 465 | 155 | UUC->UUU | Phe->Phe | TTGTTAATTAATTTCTGGTT | C | ACACGACTTCAGGCAGATAA | 0.999 |
| nad5 | 494 | 165 | ACA->AUA | Thr->Ile | TCAGGCAGATAAAGCAGCTA | C | AAAAGCTATGCCTGTAAATC | 1 |
| nad5 | 506 | 169 | CCU->CUU | Pro->Leu | AGCAGCTACAAAAGCTATGC | C | TGTAAATCGAGTAGGTGATT | 0.997 |
| nad5 | 539 | 180 | CCU->CUU | Pro->Leu | AGGTGATTTTGGATTAGCTC | C | TGGGATTTCGGGTCGTTTTA | 1 |
| nad5 | 548 | 183 | UCG->UUG | Ser->Leu | TGGATTAGCTCCTGGGATTT | C | GGGTCGTTTTACTCTATTTC | 0.998 |
| nad5 | 553 | 185 | CGU->UGU | Arg->Cys | TAGCTCCTGGGATTTCGGGT | C | GTTTTACTCTATTTCAAACA | 0.996 |
| nad5 | 581 | 194 | UCU->UUU | Ser->Phe | TCTATTTCAAACAGTAGACT | C | TTCAACTATTTTTGCTCGTG | 0.979 |
| nad5 | 629 | 210 | UCU->UUU | Ser->Phe | CCCAAGAAATTCATGGATTT | C | TCGCAATATGAGATTGAATG | 0.977 |
| nad5 | 631 | 211 | CGC->UGC | Arg->Cys | CAAGAAATTCATGGATTTCT | C | GCAATATGAGATTGAATGCC | 0.98 |
| nad5 | 713 | 238 | UCG->UUG | Ser->Leu | GAAATCTGCACAGATAGGAT | C | GCATACTCGGTCACCCGATG | 0.988 |
| nad5 | 721 | 241 | CGG->UGG | Arg->Trp | CACAGATAGGATCGCATACT | C | GGTCACCCGATGCAATGGAG | 0.987 |
| nad5 | 725 | 242 | UCA->UUA | Ser->Leu | GATAGGATCGCATACTCGGT | C | ACCCGATGCAATGGAGGGCC | 0.999 |
| nad5 | 835 | 279 | CCA->UCA | Pro->Ser | GCTCCCCTTTATTTGAATAC | C | CACCTACGGCTTTGATTGTT | 1 |
| nad5 | 863 | 288 | UCU->UUU | Ser->Phe | GGCTTTGATTGTTATTACTT | C | TGCAGGAGCTATGACGTCAT | 1 |
| nad5 | 1184 | 395 | CCA->CUA | Pro->Leu | TCTAATTGGCTTTCCTTTTC | C | AACTGGATTTTATTCCAAAG | 1 |
| nad5 | 1400 | 467 | UCA->UUA | Ser->Leu | CATTCCTATGGCCATTCCTT | C | AATACTTCTGGCTCTCGGGA | 1 |
| nad5 | 1490 | 497 | CCC->CUC | Pro->Leu | CAATTTTTGGGCCAATTCCC | C | CTTCGTACTACCAAAAAATG | 0.999 |
| nad5 | 1550 | 517 | ACA->AUA | Thr->Ile | GTTTGCTGCTCCAACCATTA | C | AAAACTAATACCTATTCTCT | 0.929 |
| nad5 | 1580 | 527 | UCA->UUA | Ser->Leu | ACCTATTCTCTTTAGTACTT | C | AGGTGCTTCTCTGGCGTATA | 0.978 |
| nad5 | 1589 | 530 | UCU->UUU | Ser->Phe | CTTTAGTACTTCAGGTGCTT | C | TCTGGCGTATAATGTAAATC | 0.966 |
| nad5 | 1610 | 537 | CCU->CUU | Pro->Leu | TCTGGCGTATAATGTAAATC | C | TGTAGCGGATAAATTCCAAC | 0.919 |
| nad5 | 1859 | 620 | UCU->UUU | Ser->Phe | AAGTCAACTTCAAAGTGGAT | C | TGTTTATCATTATGCCTTTG | 0.998 |
| nad5 | 1895 | 632 | UCA->UUA | Ser->Leu | CTTTGCAATGCTACTTGGTT | C | AACTCCATTTGTGACCTTTT | 0.961 |
| nad5 | 1901 | 634 | CCA->CUA | Pro->Leu | AATGCTACTTGGTTCAACTC | C | ATTTGTGACCTTTTCTCGTA | 0.954 |
| nad5 | 1916 | 639 | UCU->UUU | Ser->Phe | AACTCCATTTGTGACCTTTT | C | TCGTATGTGGGACTCTCTAT | 0.998 |
| nad5 | 1918 | 640 | CGU->UGU | Arg->Cys | CTCCATTTGTGACCTTTTCT | C | GTATGTGGGACTCTCTATCT | 0.997 |
| nad6 | 17 | 6 | UCG->UUG | Ser->Leu | TACCATGATACTTTCTGTTT | C | GTCGAGCCCCGCTTTGGTCT | 0.996 |
| nad6 | 26 | 9 | CCC->CUC | Pro->Leu | ACTTTCTGTTTCGTCGAGCC | C | CGCTTTGGTCTCTGGTTTGA | 0.988 |
| nad6 | 88 | 30 | CCC->UUC | Pro->Phe | CGGTACATTCTGTTTTGTTT | C | CCATCCCAGTCTTTCGCGAC | 0.974 |
| nad6 | 89 | 30 | CCC->UUC | Pro->Phe | GGTACATTCTGTTTTGTTTC | C | CATCCCAGTCTTTCGCGACA | 0.999 |
| nad6 | 95 | 32 | CCA->CUA | Pro->Leu | TTCTGTTTTGTTTCCCATCC | C | AGTCTTTCGCGACACTTCTG | 0.998 |
| nad6 | 103 | 35 | CGC->UGC | Arg->Cys | TGTTTCCCATCCCAGTCTTT | C | GCGACACTTCTGGTTTACTT | 0.999 |
| nad6 | 146 | 49 | UCC->UUC | Ser->Phe | TTTGTTAGGTCTCGACTTCT | C | CGCTATGATCTCCCCAGTAG | 1 |
| nad6 | 158 | 53 | UCC->UUC | Ser->Phe | CGACTTCTCCGCTATGATCT | C | CCCAGTAGTTCATATAGGAG | 0.998 |
| nad6 | 161 | 54 | CCA->CUA | Pro->Leu | CTTCTCCGCTATGATCTCCC | C | AGTAGTTCATATAGGAGCTA | 0.996 |
| nad6 | 169 | 57 | CAU->UAU | His->Tyr | CTATGATCTCCCCAGTAGTT | C | ATATAGGAGCTATTGCCGTT | 0.998 |
| nad6 | 191 | 64 | UCA->UUA | Ser->Leu | TATAGGAGCTATTGCCGTTT | C | ATTCCTATTCGTGGTTATGA | 1 |
| nad6 | 289 | 97 | CUU->UUU | Leu->Phe | GTGGTATTATTGGACTGATC | C | TTTGGTGGGAAATGTTCTTC | 0.999 |
| nad6 | 379 | 127 | CAU->UAU | His->Tyr | CCTCTCTGAGATATACGGTT | C | ATGCCGGAAAGGTACGAAGT | 1 |
| nad7 | 44 | 15 | UCC->UUU | Ser->Phe | AAAGAATTTTACTTTGAATT | C | CGGACCTCAACATCCTGCTG | 0.987 |
| nad7 | 45 | 15 | UCC->UUU | Ser->Phe | AAGAATTTTACTTTGAATTC | C | GGACCTCAACATCCTGCTGC | 0.998 |
| nad7 | 77 | 26 | UCA->UUA | Ser->Leu | TCCTGCTGCTCATGGTGTTT | C | ACGATCAGTATTGGAAATGA | 0.991 |
| nad7 | 83 | 28 | UCA->UUA | Ser->Leu | TGCTCATGGTGTTTCACGAT | C | AGTATTGGAAATGAACGGAG | 1 |
| nad7 | 137 | 46 | UCA->UUA | Ser->Leu | TGCGGAACCACATATTGGAT | C | ACTCCATAGAGGGACTGAGA | 0.999 |
| nad7 | 209 | 70 | UCA->UUA | Ser->Leu | TTTACCTTATTTTGATCGTT | C | AGACTATGTTTCTATGATGG | 0.999 |
| nad7 | 244 | 82 | CAU->UAU | His->Tyr | TGATGGCCCAAGAACACGCT | C | ATTCTTCAGCCGTAGAGAGA | 0.999 |
| nad7 | 251 | 84 | UCA->UUA | Ser->Leu | CCAAGAACACGCTCATTCTT | C | AGCCGTAGAGAGACTTTTTA | 0.982 |
| nad7 | 316 | 106 | CGU->UGU | Arg->Cys | AATATATACGAGTGTTATTC | C | GTGAAATAACTCGAATTTCA | 0.997 |
| nad7 | 335 | 112 | UCA->UUA | Ser->Leu | CCGTGAAATAACTCGAATTT | C | AAATCATTCACTTGCTTCAA | 0.998 |
| nad7 | 344 | 115 | UCA->UUA | Ser->Leu | AACTCGAATTTCAAATCATT | C | ACTTGCTTCAACTACTCATG | 0.999 |
| nad7 | 353 | 118 | UCA->UUA | Ser->Leu | TTCAAATCATTCACTTGCTT | C | AACTACTCATGCTATGGATG | 0.999 |
| nad7 | 383 | 128 | UCA->UUA | Ser->Leu | TGCTATGGATGTGGGAGCAT | C | AACTCCGTTCCTTTGGGCTT | 1 |
| nad7 | 445 | 149 | CCG->UCG | Pro->Ser | TGGAATTCTATGAAAGAGTC | C | CGGGAGCCAGGATGCATGCC | 1 |
| nad7 | 533 | 178 | UCC->UUC | Ser->Phe | ATGTCGAGATATTGATTCCT | C | CACACAACAATTTGCTTCTC | 0.998 |
| nad7 | 963 | 321 | UCC->UCU | Ser->Ser | CTTTATACAGAAGGTTTTTC | C | GTACCAGCTTCTTCTACCTA | 0.94 |
| nad9 | 92 | 31 | UCU->UUU | Ser->Phe | ATCGGAACATGGGAATAGAT | C | TTATACCAATACTGACTACC | 0.998 |
| nad9 | 113 | 38 | CCA->CUA | Pro->Leu | TTATACCAATACTGACTACC | C | ATTTCCATTGTTGTGCTTTC | 0.995 |
| nad9 | 223 | 75 | CAU->UAU | His->Tyr | AACGAAGATTTGAAGTTGTC | C | ATAATTTACTGAGTCTTCGG | 0.999 |
| nad9 | 298 | 100 | CCG->UCG | Pro->Ser | ACGAAGTAACACGAATCTCT | C | CGGTAGTAAGTCCATTTCCA | 0.995 |
| nad9 | 311 | 104 | CCA->CUA | Pro->Leu | AATCTCTCCGGTAGTAAGTC | C | ATTTCCATCAGCCGGCCGGT | 1 |
| nad9 | 328 | 110 | CGG->UGG | Arg->Trp | GTCCATTTCCATCAGCCGGC | C | GGTGGGAGCGAGAAGTATGG | 0.998 |
| nad9 | 356 | 119 | UCU->UUU | Ser->Phe | GCGAGAAGTATGGGATATGT | C | TGGTGTTTCTTCCATCAATC | 0.99 |
| nad9 | 368 | 123 | UCC->UUC | Ser->Phe | GGATATGTCTGGTGTTTCTT | C | CATCAATCATCCGGATTTAC | 0.998 |
| nad9 | 398 | 133 | UCA->UUA | Ser->Leu | TCCGGATTTACGGCGTATAT | C | AACAGATTATGGTTTCGAGG | 0.974 |
| nad9 | 539 | 180 | UCU->UUU | Ser->Phe | AGAATTTCGCTATTTTGATT | C | TGCTAGTCCTTGGGAACAGC | 1 |
| rpl10 | 101 | 34 | UCA->UUA | Ser->Leu | CTCATTCCATTCCAGTGGCT | C | AACCAGTAACCAATGGCGAA | 0.989 |
| rpl10 | 134 | 45 | CCA->CUA | Pro->Leu | ATGGCGAAAACTCAAAAATC | C | ATGGTTTCCCGGTAGAACCC | 0.994 |
| rpl16 | 35 | 12 | UCU->UUU | Ser->Phe | ACGTACGAAATATAGTAAAT | C | TAGTAAAGGCAGATGTAGTA | 0.947 |
| rpl16 | 61 | 21 | CGC->UGC | Arg->Cys | AAGGCAGATGTAGTAGGGGT | C | GCGAACCGGACGGTACACAA | 0.999 |
| rpl16 | 104 | 35 | ACC->AUU | Thr->Ile | TGGTTTTGGAAGATATGGCA | C | CAAAAGTTGTAGGGCTGGTC | 0.998 |
| rpl16 | 105 | 35 | ACC->AUU | Thr->Ile | GGTTTTGGAAGATATGGCAC | C | AAAAGTTGTAGGGCTGGTCG | 0.993 |
| rpl16 | 164 | 55 | ACA->AUA | Thr->Ile | TGAAGCAGCGCGTCGGGCTA | C | AATCGGACACTTCCATCGTG | 0.999 |
| rpl16 | 232 | 78 | CUC->UUC | Leu->Phe | GTAAGATATGGGTAAGAGTT | C | TCGCTGATCTTCCTATTACG | 0.974 |
| rpl16 | 321 | 107 | UCC->UCU | Ser->Ser | GGTTGGATTGCTCGTGTGTC | C | ACGGGACAAATCCCATTTGA | 0.903 |
| rpl16 | 335 | 112 | CCA->CUA | Pro->Leu | TGTGTCCACGGGACAAATCC | C | ATTTGAAATGGATGGTGTGA | 0.999 |
| rpl16 | 401 | 134 | CCA->CUA | Pro->Leu | TACATTAGCGGCGCATAAAC | C | ATGTTCGTCAACCAAGTTTC | 0.992 |
| rpl16 | 407 | 136 | UCG->UUG | Ser->Leu | AGCGGCGCATAAACCATGTT | C | GTCAACCAAGTTTCTTCAGT | 0.99 |
| rpl5 | 35 | 12 | UCA->UUA | Ser->Leu | TTTTCATTACGAAGATGTCT | C | ACGTCAGGATCTCTTGCTCA | 0.993 |
| rpl5 | 59 | 20 | CCG->CUG | Pro->Leu | TCAGGATCTCTTGCTCAAAC | C | GAATCACGCCAACGTTATGG | 0.995 |
| rpl5 | 64 | 22 | CAC->UAC | His->Tyr | ATCTCTTGCTCAAACCGAAT | C | ACGCCAACGTTATGGAAGTT | 0.966 |
| rpl5 | 92 | 31 | UCG->UUG | Ser->Leu | CGTTATGGAAGTTCCTGGAT | C | GTGTGAAATCAGATTAGTAC | 0.998 |
| rpl5 | 166 | 56 | CCG->UCG | Pro->Ser | GAAAATTGGCTATGGAGATT | C | CGCGCGGTCAGAGATTCATA | 0.98 |
| rpl5 | 169 | 57 | CGC->UGC | Arg->Cys | AATTGGCTATGGAGATTCCG | C | GCGGTCAGAGATTCATACAG | 0.998 |
| rpl5 | 239 | 80 | UCC->UUC | Ser->Phe | AAAGTCGTTTCGATCCTGGT | C | CGAAAAAGACACAGGCAGTG | 0.982 |
| rpl5 | 320 | 107 | UCG->UUG | Ser->Leu | TCATTTTTTGGTCAGAATCT | C | GACAGTAATGTCTATGTTAG | 0.952 |
| rpl5 | 367 | 123 | CCC->UCC | Pro->Ser | CGGTCGAAATACGGGAAAAC | C | CCATTCAATTCTCGATGGAA | 1 |
| rpl5 | 503 | 168 | CCA->CUA | Pro->Leu | GACAAAAGATGAGACTTTAC | C | ACCGTGGAGCGGCTTTTTGC | 0.991 |
| rpl5 | 506 | 169 | CCG->CUG | Pro->Leu | AAAAGATGAGACTTTACCAC | C | GTGGAGCGGCTTTTTGCTCA | 0.981 |
| rps12 | 71 | 24 | UCG->UUG | Ser->Leu | CACGGACCGTACTCGAGCCT | C | GGATCAATGTCCCCAGAAGC | 0.998 |
| rps12 | 196 | 66 | CAC->UAC | His->Tyr | ATCGACATGATATATTTGCT | C | ACATTCCAGGCGAAGGTCAT | 0.999 |
| rps12 | 232 | 78 | CCC->UCC | Pro->Ser | GTCATAATTTGCAGGAACAT | C | CCATAGTATTAGTCAGAGGA | 0.994 |
| rps12 | 269 | 90 | UCG->UUG | Ser->Leu | AGGAGGTAGAGTGAAAGATT | C | GCCAGGTGTGAAATCCCATC | 0.995 |
| rps12 | 284 | 95 | UCC->UUC | Ser->Phe | AGATTCGCCAGGTGTGAAAT | C | CCATCGTATTCGAGGAGTCA | 0.998 |
| rps12 | 289 | 97 | CGU->UGU | Arg->Cys | CGCCAGGTGTGAAATCCCAT | C | GTATTCGAGGAGTCAAGGAT | 1 |
| rps13 | 26 | 9 | UCA->UUA | Ser->Leu | ATATATTTCAGGAGCTAGAT | C | AGTTCCCGATGAACAAGTAA | 0.999 |
| rps13 | 56 | 19 | UCA->UUA | Ser->Leu | TGAACAAGTAAGAATTTCCT | C | AACAAAAATGGATGGAATTG | 0.964 |
| rps13 | 100 | 34 | CGU->UGU | Arg->Cys | CTAAAAAAGCCATTCAGCTT | C | GTTATCGATTAGGTCTCAGT | 0.984 |
| rps13 | 256 | 86 | CCU->UUU | Pro->Phe | AACGATTAATTTATATTTCT | C | CTTATCGTGGAATTCGTCAT | 0.992 |
| rps13 | 257 | 86 | CCU->UUU | Pro->Phe | ACGATTAATTTATATTTCTC | C | TTATCGTGGAATTCGTCATC | 0.949 |
| rps13 | 287 | 96 | UCG->UUG | Ser->Leu | AATTCGTCATCAAGATGGAT | C | GCCCTTACGCGGTCAACGAA | 0.999 |
| rps19 | 116 | 39 | UCG->UUG | Ser->Leu | GTCACGTAGATCTTCTATTT | C | GCCGGAATTCGTTGATTGCT | 0.981 |
| rps19 | 163 | 55 | CCU->UUU | Pro->Phe | TCATTTACAATGGAAAAACT | C | CTCTTCGTTGTAAGATTACT | 0.97 |
| rps19 | 164 | 55 | CCU->UUU | Pro->Phe | CATTTACAATGGAAAAACTC | C | TCTTCGTTGTAAGATTACTG | 0.983 |
| rps2 | 179 | 60 | CCC->CUC | Pro->Leu | TCTTCATTTTATAGGATCTC | C | CATTCGTCAAAAAGGCCGTT | 0.944 |
| rps2 | 449 | 150 | GCA->GUA | Ala->Val | TCGATCACAAATACCTATTG | C | ATCCTTAGTTGATTCTACGA | 0.961 |
| rps2 | 520 | 174 | CCU->UCU | Pro->Ser | ATCCCATTCCAGCAAATGAT | C | CTATACAGTTCGTATATCTA | 0.986 |
| rps2 | 544 | 182 | CGU->UGU | Arg->Cys | TACAGTTCGTATATCTATTT | C | GTCATTCGATCACGAAAACT | 0.964 |
| rps3 | 58 | 20 | CCA->UCA | Pro->Ser | ATCTGAATCGTAGTTCAGAT | C | CAAGTCGGTTCAGCGATTAT | 0.996 |
| rps3 | 64 | 22 | CGG->UGG | Arg->Trp | ATCGTAGTTCAGATCCAAGT | C | GGTTCAGCGATTATTATTAT | 0.997 |
| rps3 | 69 | 23 | UUC->UUU | Phe->Phe | AGTTCAGATCCAAGTCGGTT | C | AGCGATTATTATTATGGTAA | 0.936 |
| rps3 | 92 | 31 | UCA->UUA | Ser->Leu | CGATTATTATTATGGTAAAT | C | AGTGTATCAAGATGTCAATC | 0.995 |
| rps3 | 512 | 171 | UCA->UUA | Ser->Leu | AAAGAAGAATTTATCTAAAT | C | ACTTCGGGTCAGCGGGGCCT | 0.933 |
| rps3 | 995 | 332 | CCA->CUA | Pro->Leu | CGGTGCTACCTTTTTCTTTC | C | AAGGGATGGGGTTGGGGTTA | 0.999 |
| rps3 | 1355 | 452 | CCG->CUG | Pro->Leu | TCAAGACATCTCTTTTCAAC | C | GAGGAATAATAAAAGATCAT | 0.998 |
| rps3 | 1519 | 507 | CGU->UGU | Arg->Cys | GAAAGTTTGGAAAAACATCT | C | GTAATGTATTTAACCATAAA | 0.973 |
| rps3 | 1552 | 518 | CCU->UCU | Pro->Ser | ACCATAAAATCGATTATGCT | C | CTGCGGAAGTCTCTACTCGT | 0.94 |
| rps3 | 1583 | 528 | UCA->UUA | Ser->Leu | CTCTACTCGTTACGGAATTT | C | AGGTGTCAAAGTGCGGATCT | 0.997 |
| rps3 | 1597 | 533 | CGG->UGG | Arg->Trp | GAATTTCAGGTGTCAAAGTG | C | GGATCTCATATACTCAAAAT | 1 |
| sdh4 | 29 | 10 | UCG->UUG | Ser->Leu | GGCATTTTGTAGACGTGGTT | C | GGTTATTCCCATTTGTCTCT | 1 |
| sdh4 | 39 | 13 | CCC->CCU | Pro->Pro | AGACGTGGTTCGGTTATTCC | C | ATTTGTCTCTATCTATTGGT | 1 |
| sdh4 | 156 | 52 | UUC->UUU | Phe->Phe | TTTATTAAAAATGACTACTT | C | CATCTCTGTATTCTGGCTAG | 0.971 |
