# Supplementary material for: Structure and multipartite genome architecture of the mitochondrial genome in the endangered medicinal plant Fritillaria taipaiensis P. Y. Li
Source: Front Syst Biol. 2026 Apr 22;6:1708877. doi: 10.3389/fsysb.2026.1708877 (PMC13143598; doi:10.3389/fsysb.2026.1708877)

**Supplemental Figures**

**IGV visualization of short-read (Illumina) and long-read (Nanopore) mapping across the MTPT1-10 region in the mitochondrial genome.**

**
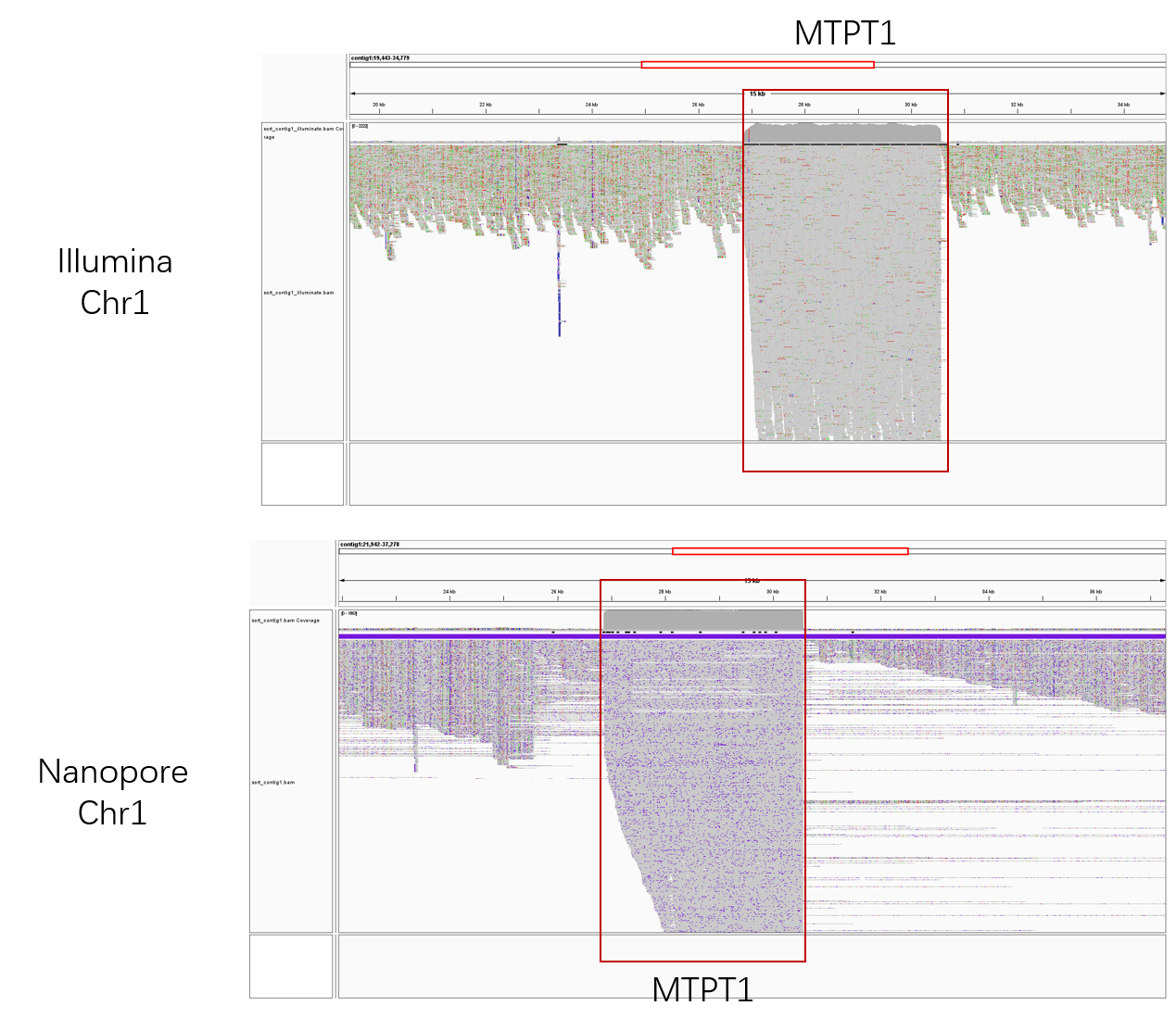
**


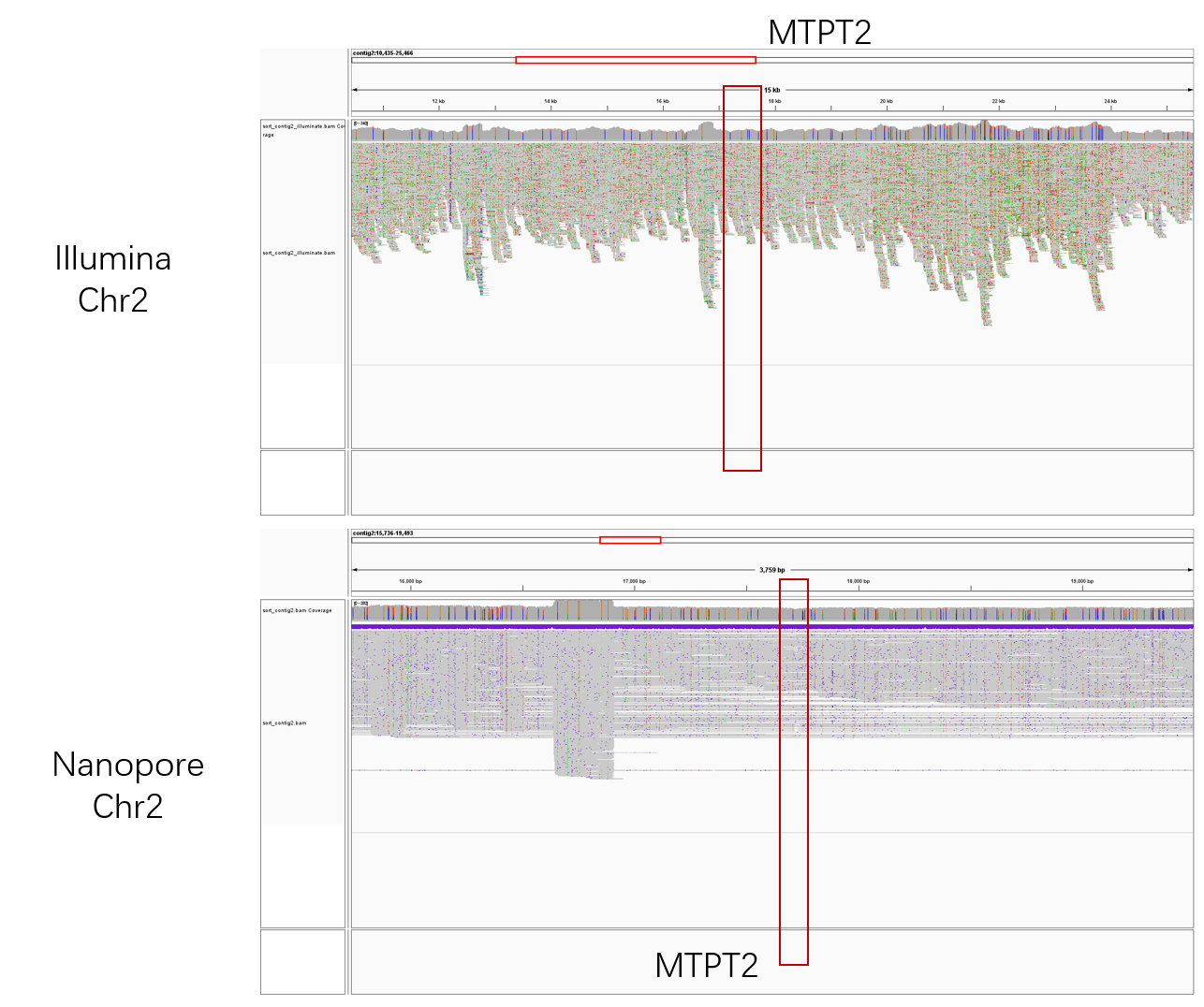


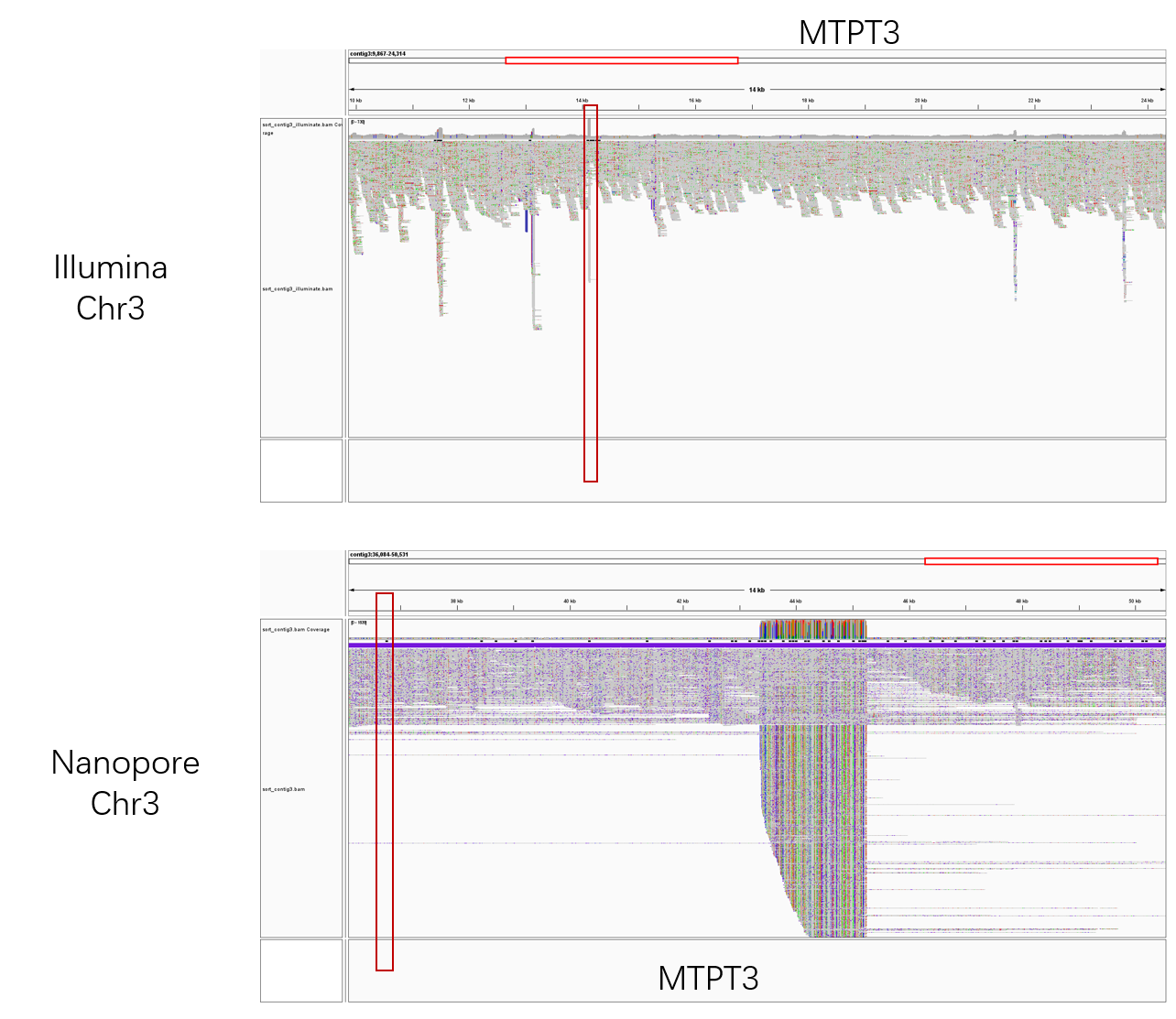


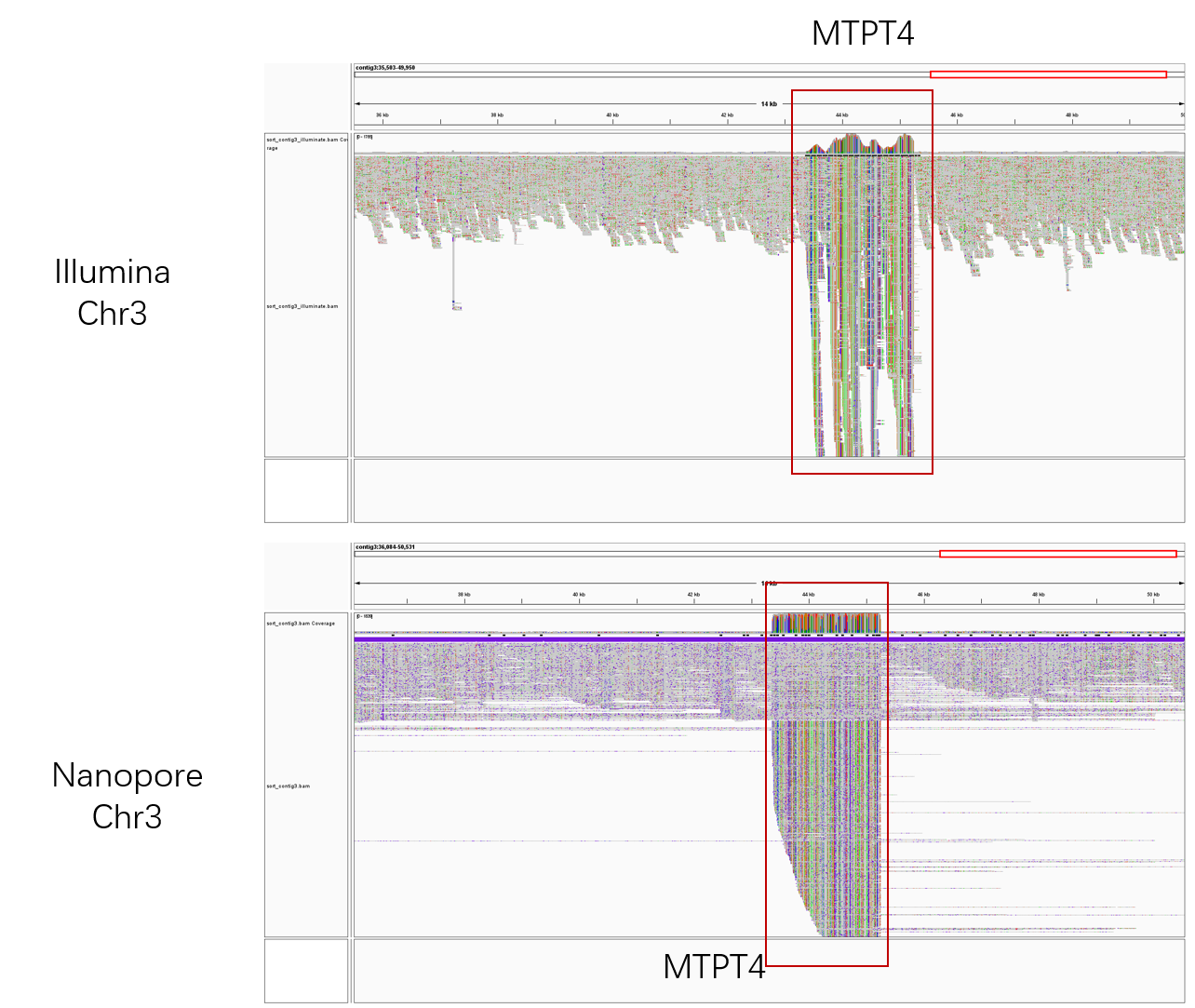


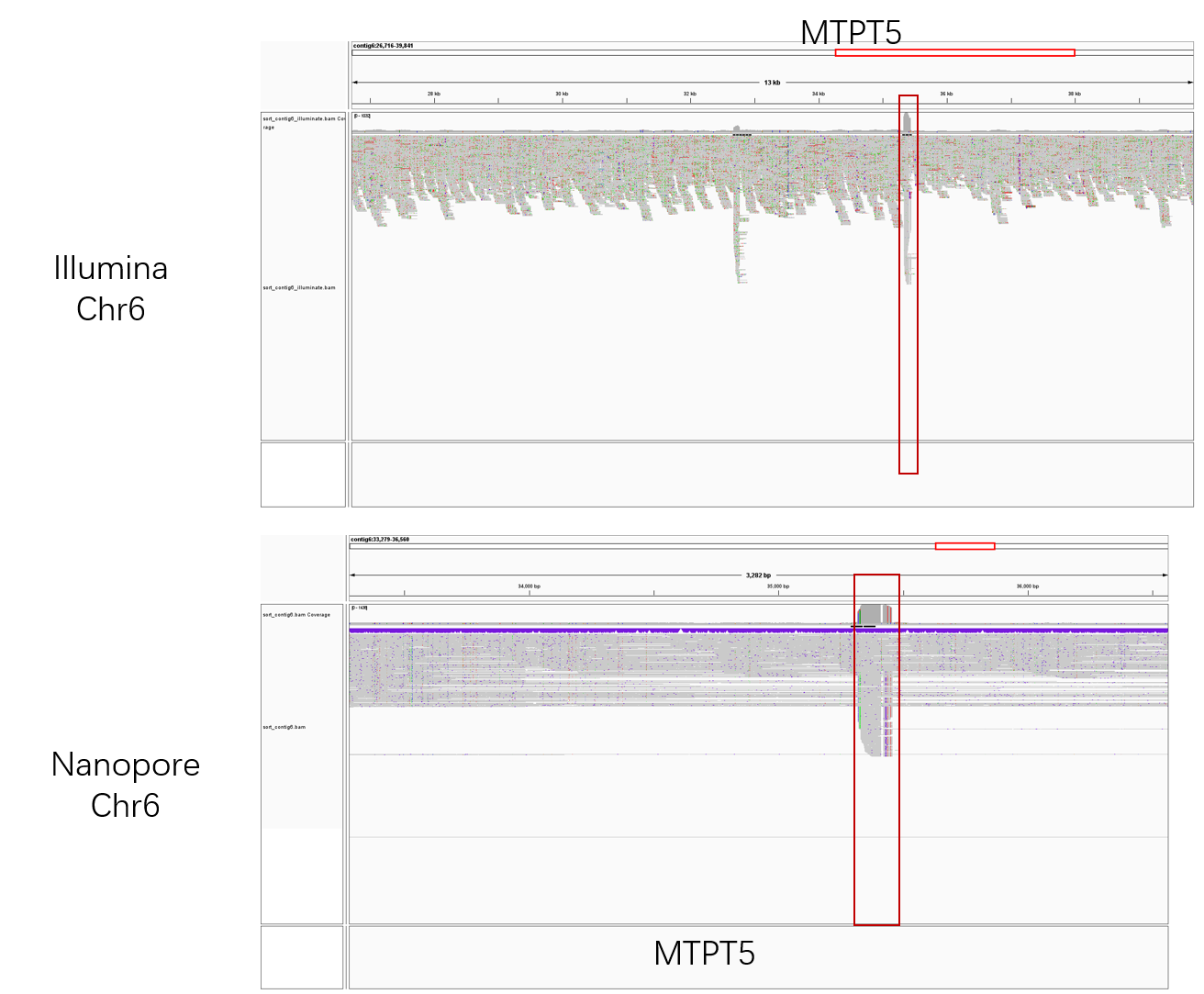


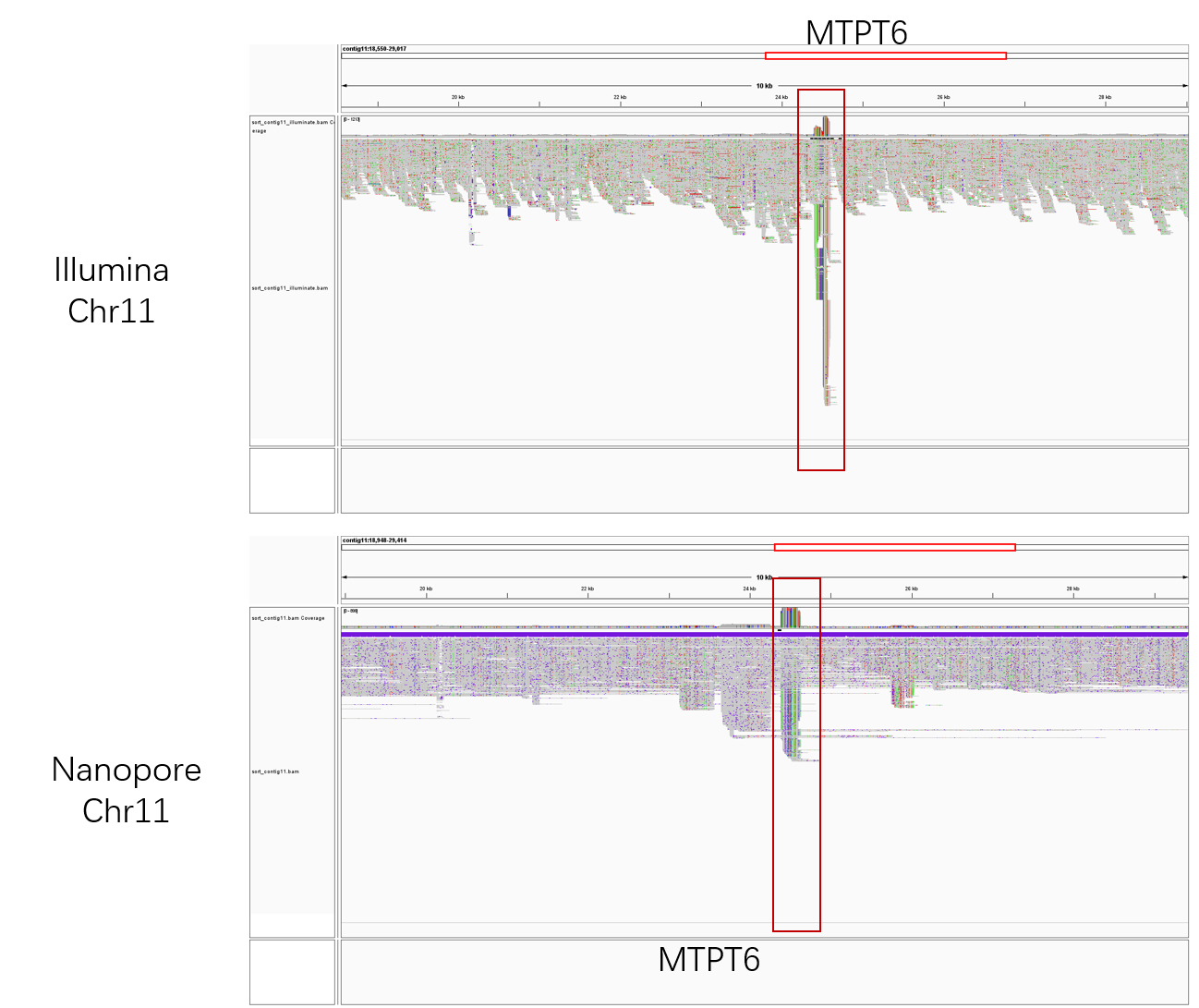


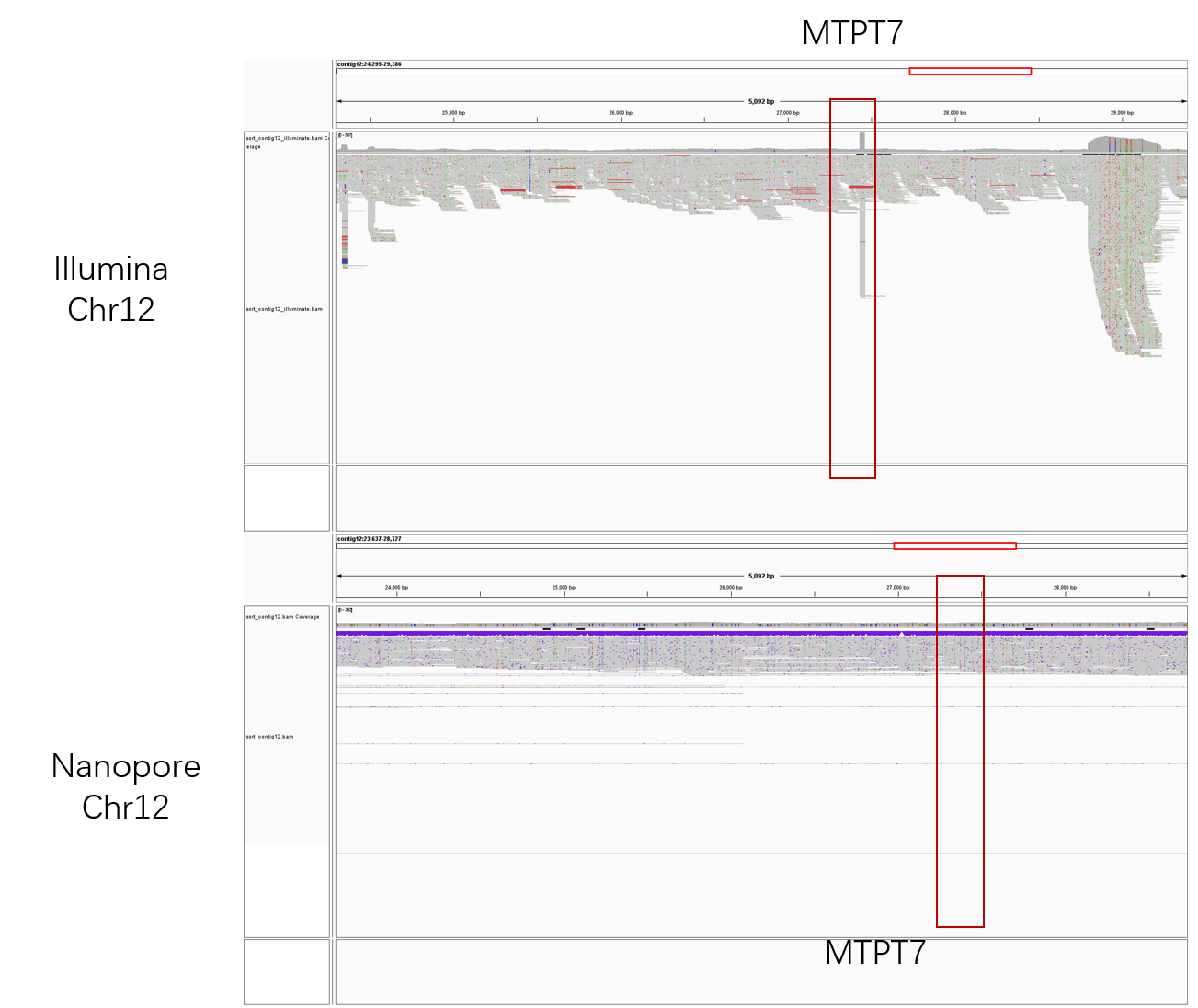


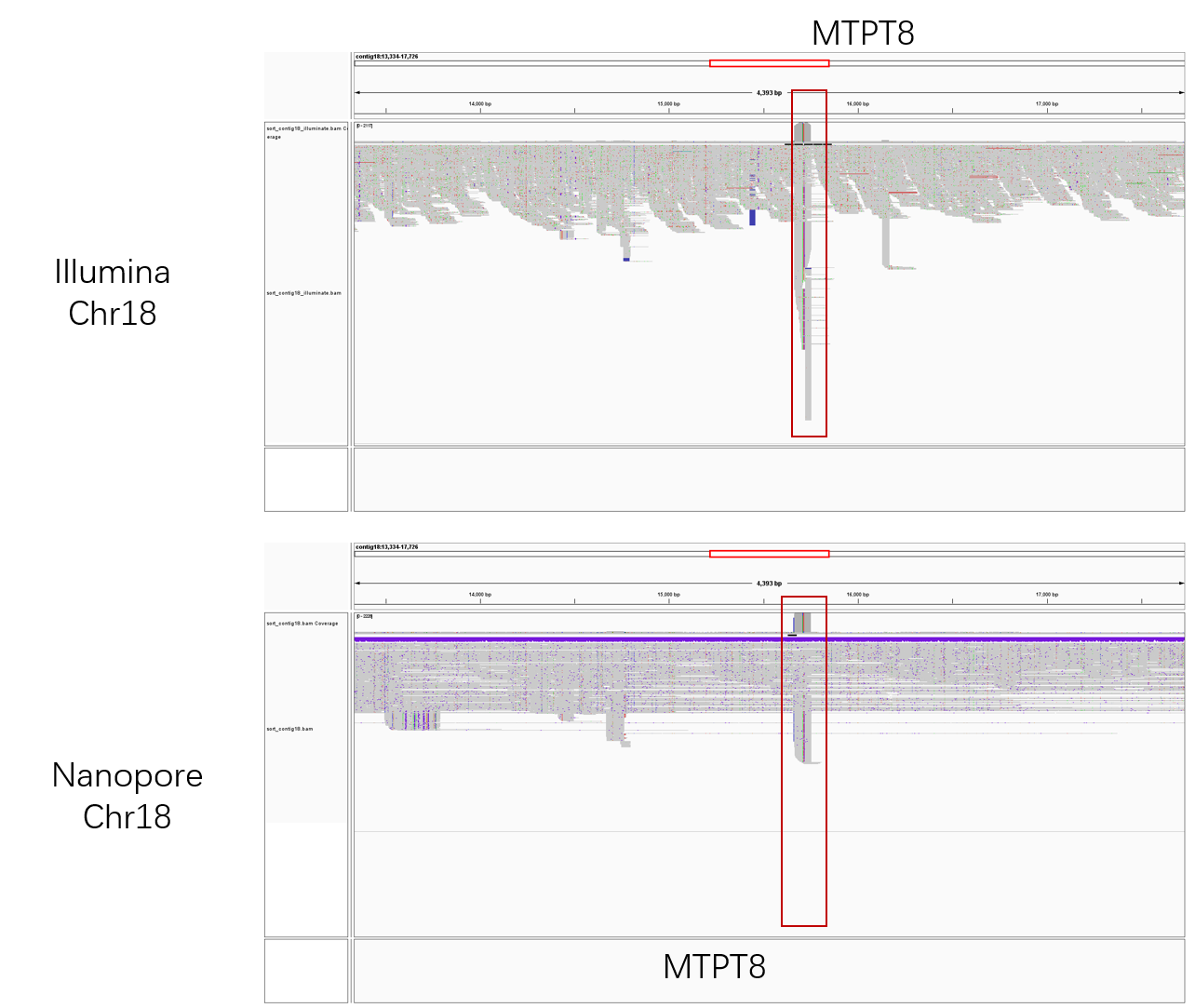


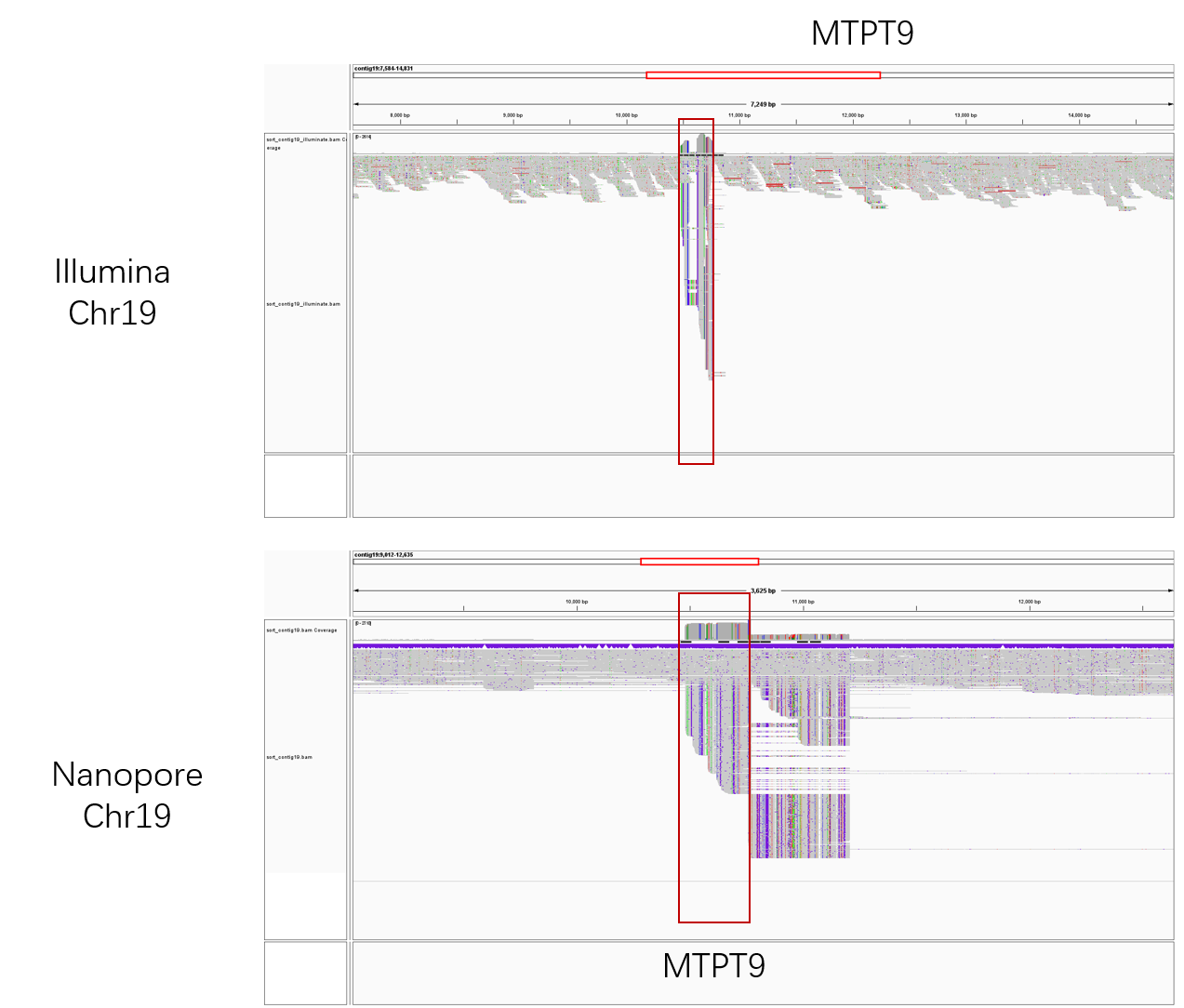


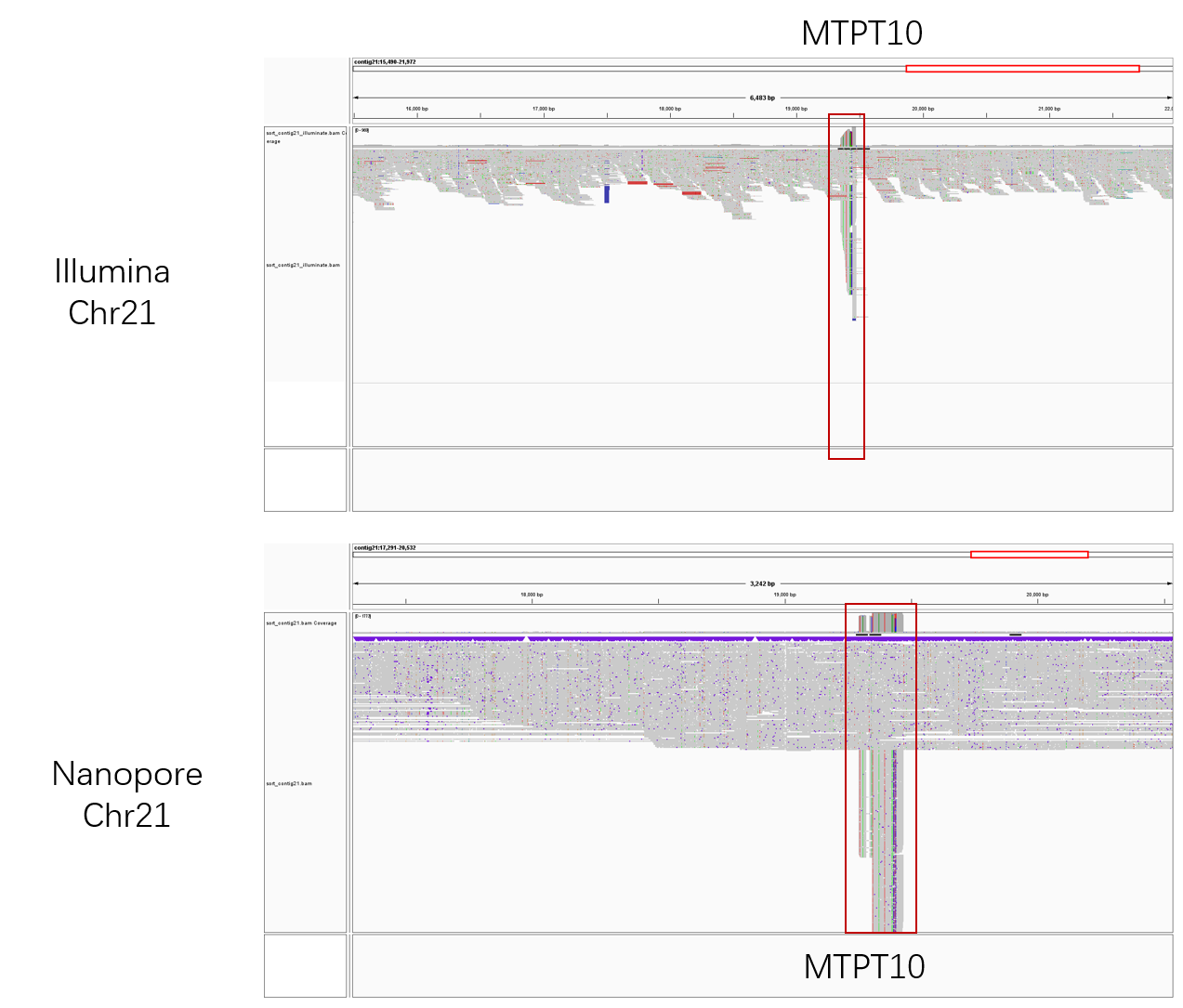

Supplement: Supplementary file 4 [file Supplementaryfile1.doc]
